# Linking sporadic hospital clusters during a community surge of the severe acute respiratory coronavirus virus 2 (SARS-CoV-2) B.1.617.2 delta variant: The utility of whole-genome sequencing

**DOI:** 10.1017/ice.2022.106

**Published:** 2023-06

**Authors:** Liang En Wee, Karrie Kwan-Ki Ko, Edwin Philip Conceicao, Jean Xiang-Ying Sim, Nurdyana Abdul Rahman, Shireen Yan-Ling Tan, Delphine Yan-Hong Cao, Kenneth Xing-Long Chan, May Kyawt Aung, Myat Oo Aung, Yang Yong, Shalvi Arora, Molly Kue Bien How, Kwee Yuen Tan, Lai Chee Lee, Thuan Tong Tan, Limin Wijaya, James Heng Chiak Sim, Chayaporn Suphavilai, Indumathi Venkatachalam, Moi Lin Ling

**Affiliations:** 1 Department of Infectious Diseases, Singapore General Hospital, Singapore, Singapore; 2 Department of Molecular Pathology, Singapore General Hospital, Singapore, Singapore; 3 Department of Microbiology, Singapore General Hospital, Singapore, Singapore; 4 Genome Institute of Singapore, A*STAR, Singapore, Singapore; 5 Duke–NUS Medical School, Singapore, Singapore; 6 Department of Infection Prevention and Epidemiology, Singapore General Hospital, Singapore, Singapore

## Abstract

Sporadic clusters of healthcare-associated coronavirus disease 2019 (COVID-19) occurred despite intense rostered routine surveillance and a highly vaccinated healthcare worker (HCW) population, during a community surge of the severe acute respiratory coronavirus virus 2 (SARS-CoV-2) B.1.617.2 δ (delta) variant. Genomic analysis facilitated timely cluster detection and uncovered additional linkages via HCWs moving between clinical areas and among HCWs sharing a common lunch area, enabling early intervention.

Strict infection prevention measures may lower secondary attack rates of coronavirus disease 2019 (COVID-19) in healthcare facilities.^1^ However, healthcare-associated outbreaks persist with emergence of more transmissible variants, such as the severe acute respiratory coronavirus virus 2 (SARS-CoV-2) B.1.617.2 δ (delta) variant.^2,3^ Increased transmissibility and shortened incubation periods of novel SARS-CoV-2 variants^2,3^ further challenge the ability of outbreak investigation in guiding real-time exposure management given its time-consuming nature. Whole-genome sequencing (WGS) can potentially supplement the investigation of COVID-19 outbreaks in a clinically relevant timeframe.^4,5^

In Singapore, enhanced infection prevention measures initially contained nosocomial spread. The emergence of the B.1.617.2 variant in 2020, however, led to a major healthcare-associated COVID-19 outbreak.^3^ Large community outbreaks attributed to the B.1.617.2 variant provided impetus for heightened infection prevention measures, including surveillance via routine rostered testing of healthcare workers (HCWs) and patients.^6^ We utilized epidemiological investigations and WGS to provide insights into potential nosocomial COVID-19 clusters.

## Methods

### Institutional setting and study period

Our healthcare campus hosts a 1,785-bed tertiary-care hospital (mostly 5–6-bed cohort cubicles), a 545-bed community hospital, the national cardiac center and a large cancer center. Almost 13,000 HCWs work on campus. Vaccination uptake among HCWs was high, with ≥90.0% receiving 2 doses of mRNA vaccines (Pfizer/Moderna) by the end of June 2021. In contrast, 65.0% of inpatients were fully vaccinated by the end of June 2021. The study period spanned 5 months from June 21, 2021, through November 22, 2021.

### Enhanced infection prevention measures

Suspected COVID-19 cases with epidemiological risk (eg, close contact with a COVID-19 case) were admitted to negative-pressure single-bed isolation rooms, whereas patients without epidemiological risk presenting with clinical syndromes compatible with COVID-19 were isolated in reduced-density cohort cubicles in the respiratory surveillance ward until SARS-CoV-2 was excluded.^7^ HCWs donned N95 respirators in all clinical areas as a mandatory minimum; HCWs in isolation areas donned N95 respirators and disposable gloves, long-sleeved gowns, and face shields.

### Diagnosis of COVID-19 among HCWs and inpatients

All symptomatic HCWs had access to free polymerase chain reaction (PCR) testing at the staff clinic. Routine rostered testing was conducted for all asymptomatic HCWs beginning in April 2021, initially with fortnightly PCR. Given surging community transmission, HCW surveillance was stepped up to twice-weekly rapid antigen detection beginning September 29, 2021. HCWs working in high-risk inpatient areas were required to obtain confirmatory PCR testing. Inpatient screening was instituted from June 2021 via PCR testing on admission and subsequent weekly testing. HCWs and patients with significant close contact with a PCR-confirmed COVID-19 patient underwent a 14-day enhanced surveillance regimen. Patients were tested via PCR on days 1, 4, 7, and 10 and via daily rapid antigen detection until day 14. HCWs were tested via PCR on days 1 and 14 and via daily rapid antigen detection until day 14.

### Definition of healthcare-associated COVID-19

All COVID-19 inpatient cases were classified into community-onset or potentially healthcare-associated infection as follows^8^:

- Community onset: PCR positive ≤2 days after admission
- Indeterminate healthcare associated: PCR positive 3–7 days after admission
- Probable healthcare associated: PCR positive 8–14 days after admission
- Definite healthcare associated: PCR positive ≥15 days after admission

Incubation periods were defined as 1–14 days prior to positive PCR. The infectious period was defined as 2 days prior to symptom onset if symptomatic or 7 days prior to positive PCR if asymptomatic to 10 days after positive PCR. Significant patient close contact was defined as contact within 2 m of the index case for ≥15 minutes during the index case’s infectious period.^8^ For HCWs, significant close contact was defined as contact within 2 m of the index for ≥15 minutes. Significant unprotected contact was defined as not having utilized N95 respirators during a significant close contact episode and/or not having donned disposable gowns and gloves during episodes of prolonged physical contact. HCWs with significant unprotected contact were furloughed during the enhanced surveillance period.

### Cluster definition

Epidemiological clusters were defined as ≥2 COVID-19 cases among patients and HCWs in the same setting (ie, ward or workplace). These cluster outbreaks ended when no cases were diagnosed for 14 days. Genomic clusters were detected based on whole-genome similarity analysis, when sequences were ≤3 SNPs different and fell in the same branch of the genome similarity tree.^9^

### Epidemiologic and genetic analysis

Contact tracing was performed for all potentially healthcare-associated COVID-19 inpatient cases and all community-onset COVID-19 inpatient cases initially managed outside isolation areas, as well as all HCWs at work during their infective periods. Cases with a cycle threshold (Ct) value <31 were sent for WGS using the ARTIC protocol on Oxford Nanopore minION sequencers (Oxford Nanopore, Oxford, UK) to enable fortnightly turnaround.^9^

### Ethics statement

Ethics approval for outbreak investigation was not required under our institutional review board guidelines.

## Results

### Overview of cohort

Over 5 months, 1,523 (6.3%) of 24,361 admissions had COVID-19. Most were diagnosed <48 hours after admission (ie, community onset). Only 45 (3.0%) of 1,523 cases were classified as potentially healthcare associated, with 31 (68.9%) of these 45 detected >7 days after admission. Also, 12 (26.7%) of these 45 cases were reclassified as plausible community-onset cases (ie, ≤7 days of continuous admission prior to diagnosis, absence of epidemiological and/or sequencing link) (Supplementary Table 1 online). Overall, 352 HCWs with COVID-19 were identified during the study period. The odds ratio (OR) of COVID-19 acquisition among exposed inpatients, compared with exposed HCWs, was 14.8 [19.4% (27 of 139) vs 1.6% (10 of 624); 95% CI, 6.9–31.4; *P* < .001].

### Epidemiological clusters and genetic analysis

**Figure 1.**
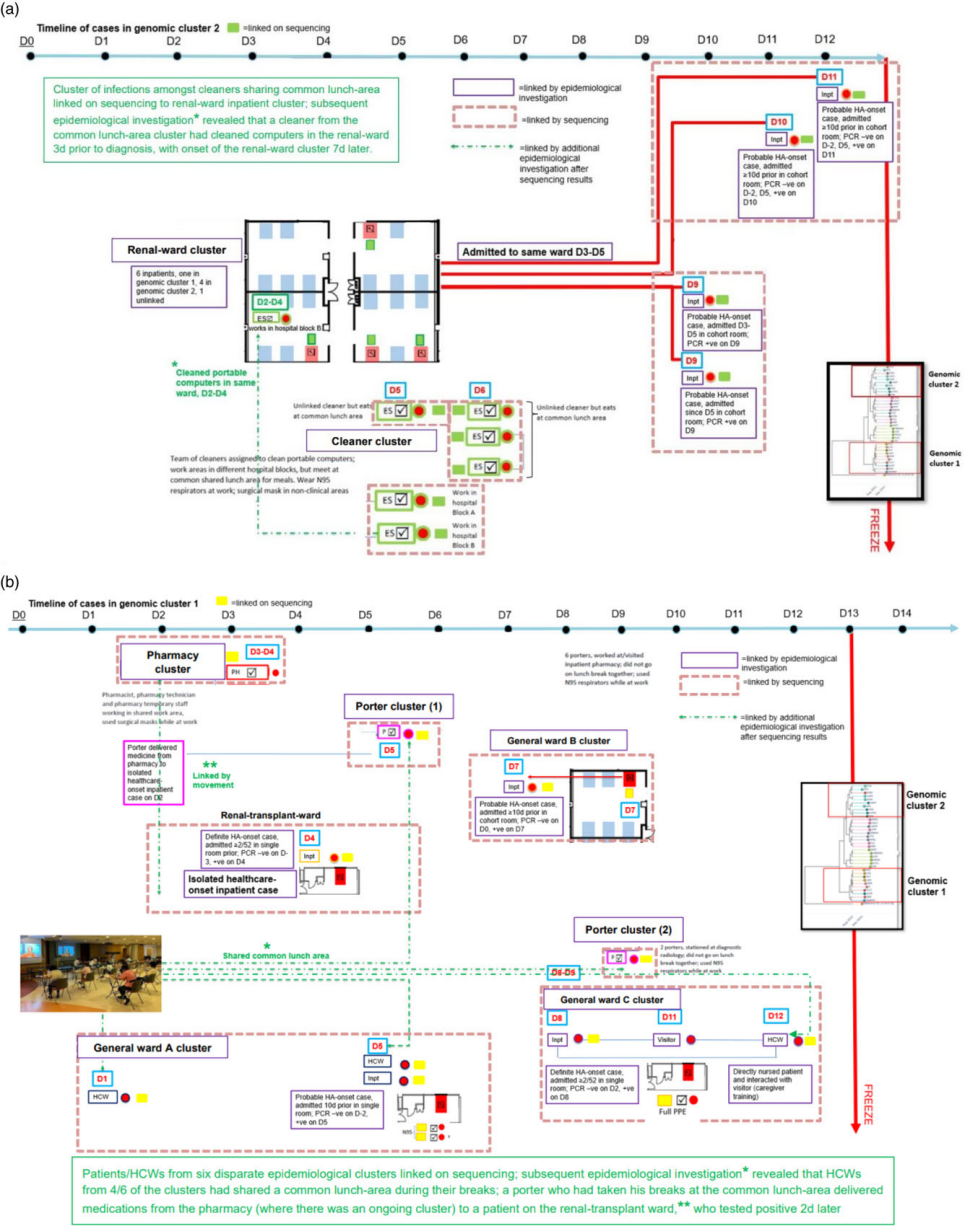
Combination of sequencing data and epidemiological investigation in linking clusters of healthcare-associated COVID-19 infection amongst staff and inpatients at a Singaporean tertiary hospital.

Samples from 488 individuals were sequenced, including 38 (84.4%) of 45 healthcare-associated inpatient cases and 203 (57.7%) of 352 HCW cases. The remaining 247 cases were community-onset cases initially managed outside isolation areas. Epidemiological investigations revealed 20 clusters (Supplementary Table 2 online). Also, 6 clusters were mixed (comprising HCWs and inpatients); 6 comprised inpatients only; and 8 occurred among HCWs. Apart from the general wards, clusters were also detected in oncology and renal wards. Contact tracing did not reveal linkages at the dialysis center (no overlap in dialysis stations or timing). Of 8 HCW clusters, 7 involved staff sharing work areas; a large cluster involved cleaners working in different areas but who mingled at a common lunch area (Supplementary Table 2 online). WGS was utilized to link previously unlinked clusters. Evidence of genomic linkage was identified between the cluster of cleaners sharing a common lunch area and the renal-ward inpatient cluster (Fig. 1a). Subsequent epidemiological investigation revealed that a cleaner in the cluster had cleaned portable computers in the renal ward (Fig. 1a). Separately, patients and HCWs from 6 disparate epidemiological clusters had genomically linked COVID-19 cases (Fig. 1b). Subsequent epidemiological investigation revealed that HCWs from the different clusters had frequented a common lunch area during their breaks (Fig. 1b). Also, 3 days prior to diagnosis, a presymptomatic porter who had taken his meals at that common lunch area delivered medication from the pharmacy (with an initial cluster of COVID-19 cases among pharmacy staff) to a patient in a single-bed room in the renal transplant ward. The patient tested positive 2 days later and was initially classified as an unlinked nosocomial COVID-19 case (>14 days after admission) because no other HCWs or patients on the renal transplant ward tested positive. The common lunch area was closed for deep cleaning with sodium hypochlorite 1,000 ppm and UV-C treatment and reopened with greater distancing between tables.

**Figure 2.**
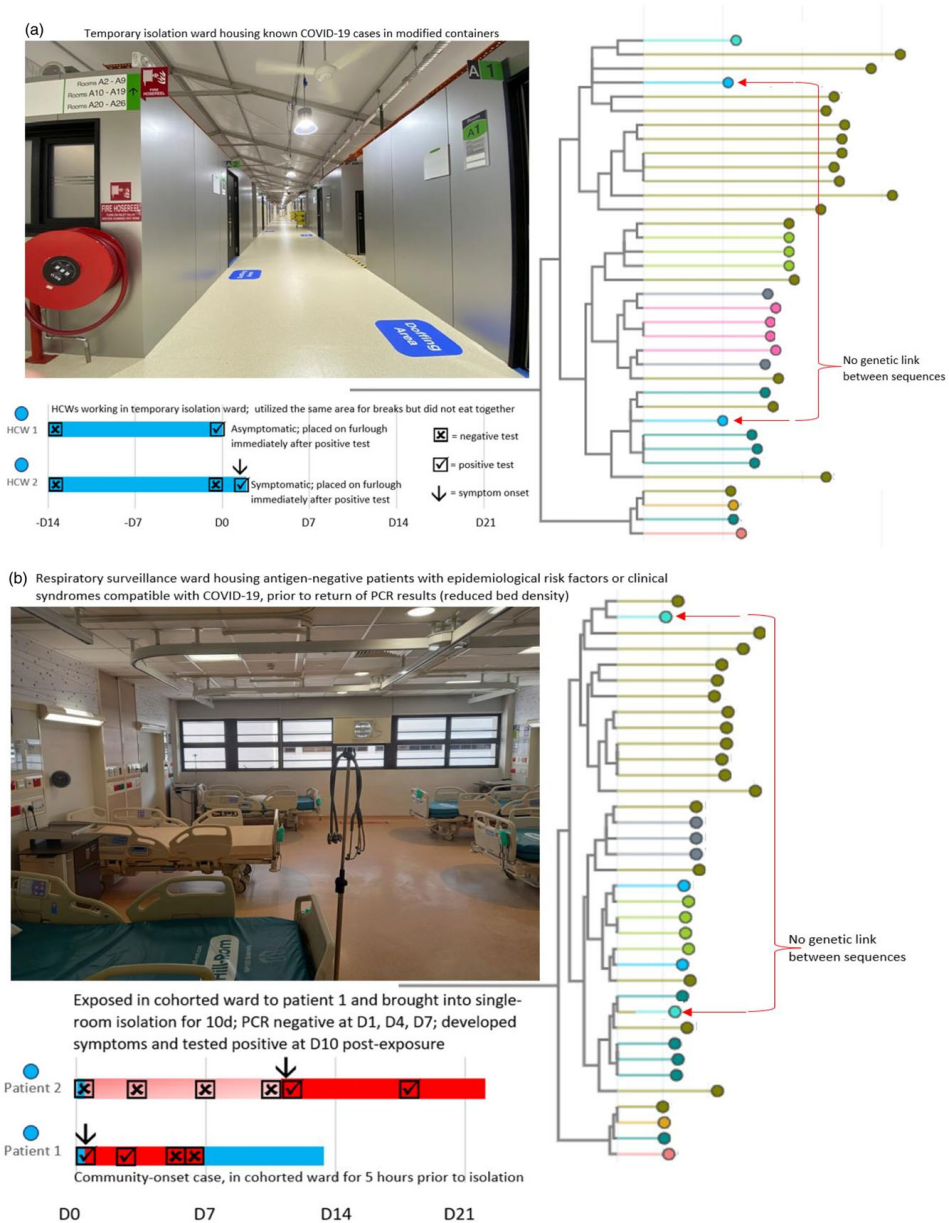
Utilisation of whole-genome-sequencing to exclude nosocomial transmission of SARS-CoV-2 in designated isolation areas.

In isolation areas, WGS was also utilized to rule out transmission. Two clusters were reported from isolation areas: 2 inpatients admitted to a respiratory surveillance ward and 2 nurses working in the container-isolation ward that housed COVID-19 patients in negative-pressure–modified containers (Supplementary Table 2 online).^10^ For the cluster in the respiratory surveillance ward, 3 patients were exposed to the index patient for 5 hours, and 1 patient tested positive upon readmission at day 9 after exposure. However, these 2 sequences were not linked (Fig. 2a), suggesting separate community acquisition. For the HCW cluster in the container-isolation ward, although both nurses utilized the same lunch area for breaks, genomic analysis confirmed that these 2 cases were not linked (Fig. 2b).

## Discussion

Genomic analysis enabled linkage of sporadic outbreaks, prompting further investigation that uncovered additional linkages via HCWs who moved between multiple wards and clinical areas but shared a common lunch area. Timely recognition of outbreaks spanning multiple areas remains challenging, given resources required to track patients and HCWs over space and time.^4,5^ WGS allowed earlier identification of transmissions based on locations other than ward of diagnosis, which facilitated subsequent intervention. The use of genomic analysis to disprove nosocomial transmission in high-risk isolation areas, despite classical epidemiological methods suggesting linkage, was crucial in maintaining staff confidence in infection prevention protocols.

This study had several limitations. Although comprehensive, sequencing was not available for all healthcare-associated infections, given difficulties with WGS at low viral loads. Additionally, these findings cannot be extrapolated to future variants with different immune escape properties.

In conclusion, sporadic healthcare-associated COVID-19 clusters occurred despite intense surveillance and enhanced infection prevention measures. Genomic analysis facilitated early cluster detection and timely intervention.

## Supplementary material

For supplementary material accompanying this paper visit https://doi.org/10.1017/ice.2022.106. click here to view supplementary material
