# Supplementary material for: Linking sporadic hospital clusters during a community surge of the severe acute respiratory coronavirus virus 2 (SARS-CoV-2) B.1.617.2 delta variant: The utility of whole-genome sequencing
Source: Infect Control Hosp Epidemiol. 2022 Apr 27;44(6):1014–8. doi: 10.1017/ice.2022.106 (PMC10262163; doi:10.1017/ice.2022.106)
Supplement: Supplementary file 1 [file S0899823X22001064sup.zip › S0899823X22001064sup001.docx]

= index case of cluster (pt)

= unlinked case

**Supplementary Table 2: Clusters of potential healthcare-associated COVID-19 infection (N=20) amongst inpatient and healthcare worker (HCW) populations within a Singaporean tertiary hospital, over a 5-month period of enhanced surveillance**

| **Location of cluster** | **Inpatient, HCW, or mixed cluster** | **Number of HCW on enhanced surveillance** | **Number of inpatients on enhanced surveillance** | **Potential community link?** | **Part of cluster on epidemiological investigation?** | **Part of cluster on genomic investigations (WGS)?** | **Spot map**  = pt with significant exposure that tested positive subsequently  = linked cases on sequencing  = vaccinated  **☑**🗷  = not fully vaccinated  🗷 |
| --- | --- | --- | --- | --- | --- | --- | --- |
| **Clusters of potential healthcare-associated COVID-19 infection involving inpatient cases on initial epidemiological investigation** | | | | | | |  |
| General ward (single-bedded) | Mixed (N=3) | 31 (fully vaccinated) | 0 | No | Yes, epi cluster 1. 1 inpatient case, 2 HCWs on the same ward | Yes; genomic cluster 1. All three cases linked genetically. | 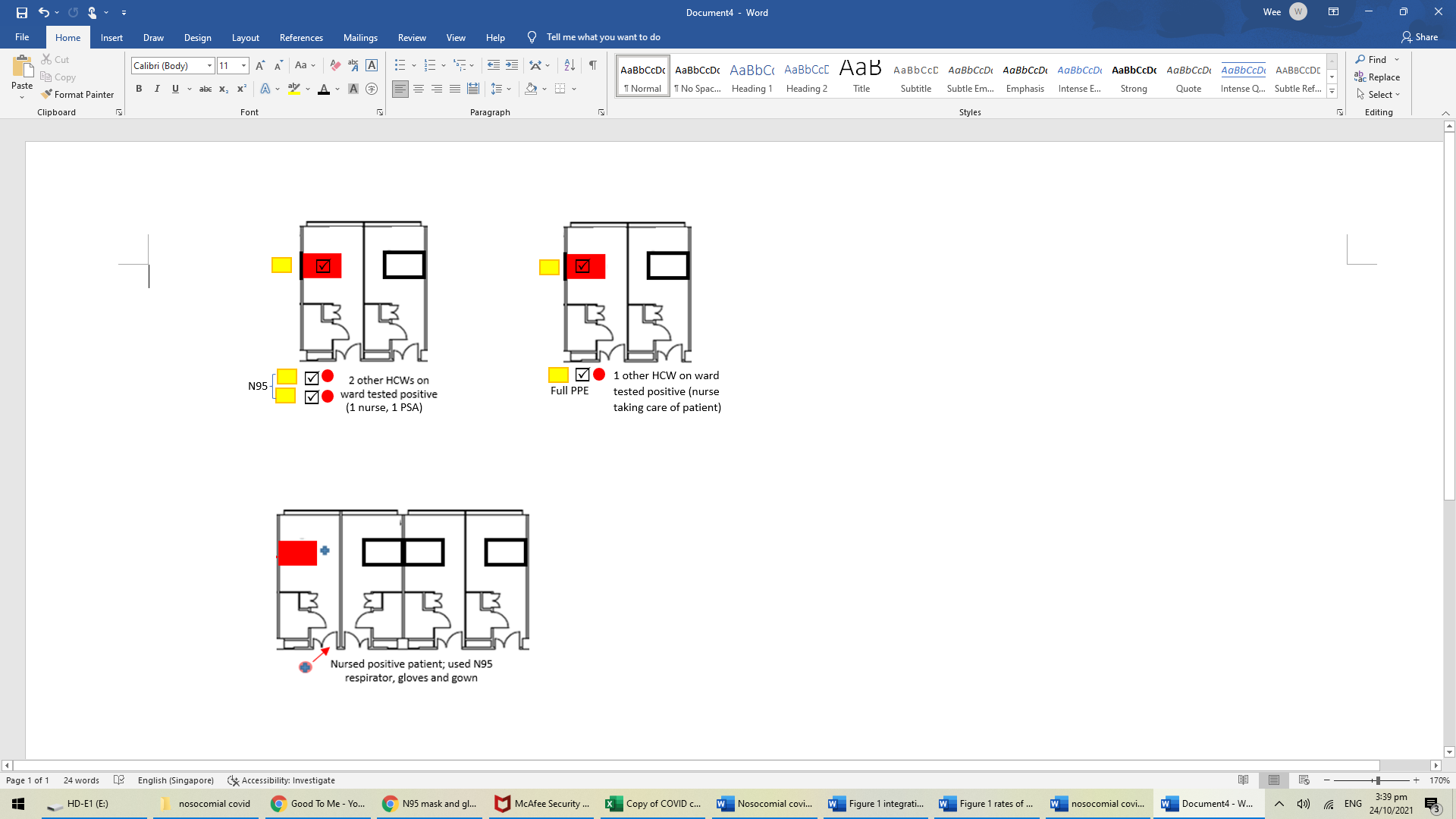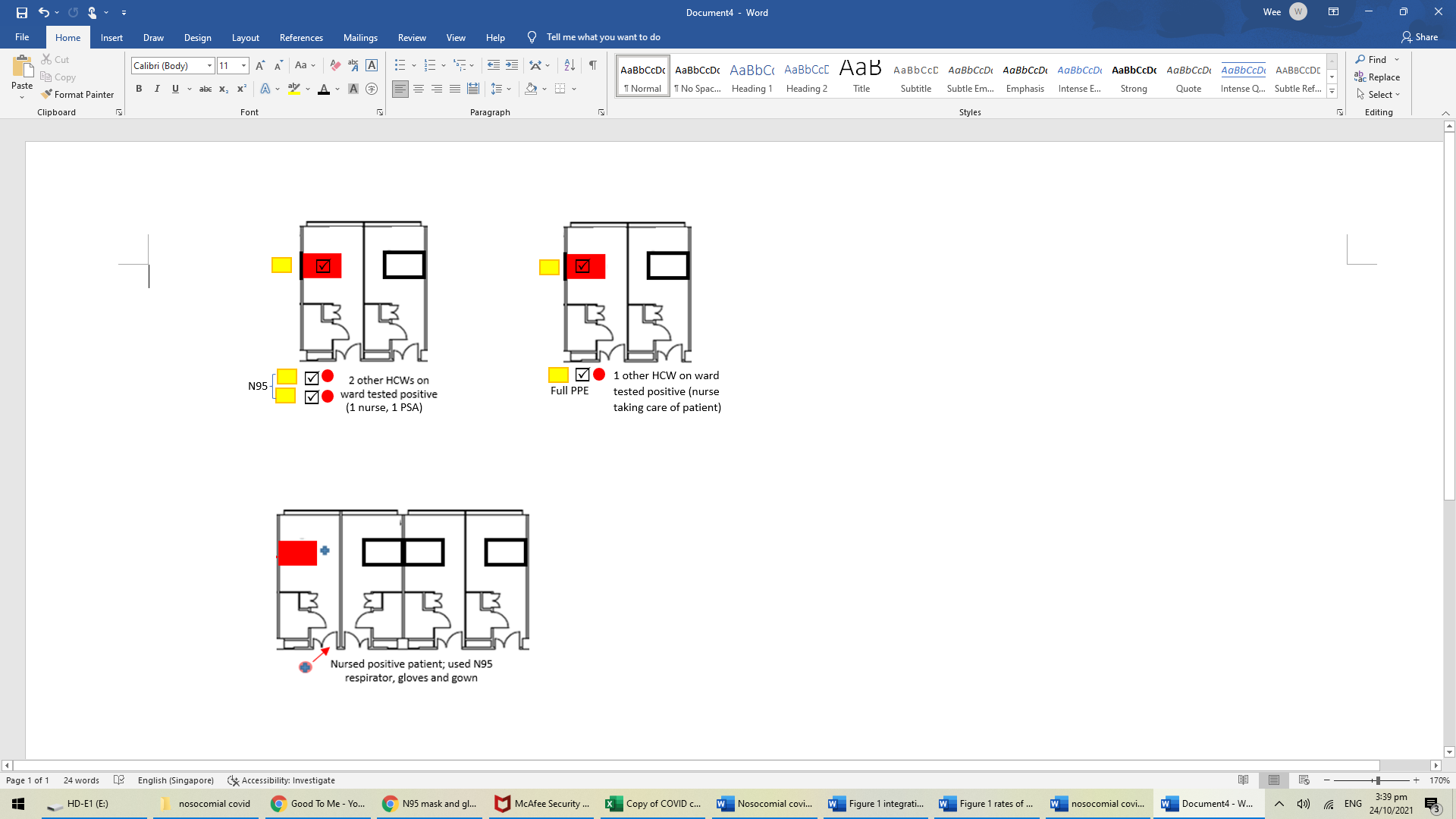 |
| General ward (single-bedded) | Mixed (N=2) | 48 (fully vaccinated) | 0 | Had C+ visitor | Yes, epi cluster 2. 1 inpatient case and 1 HCW in close-contact with the patient | Yes; genomic cluster 1. Both cases linked genetically. | 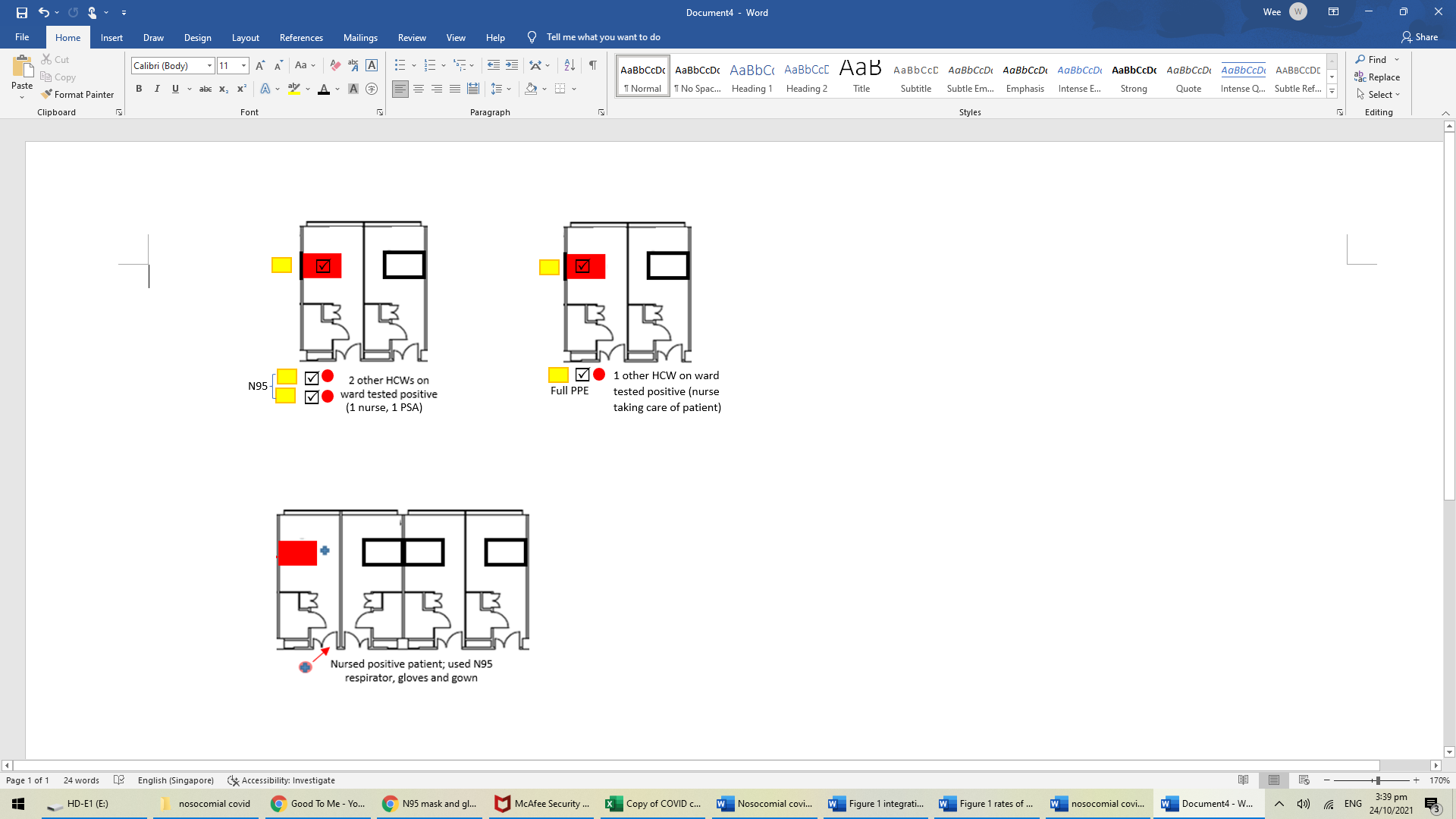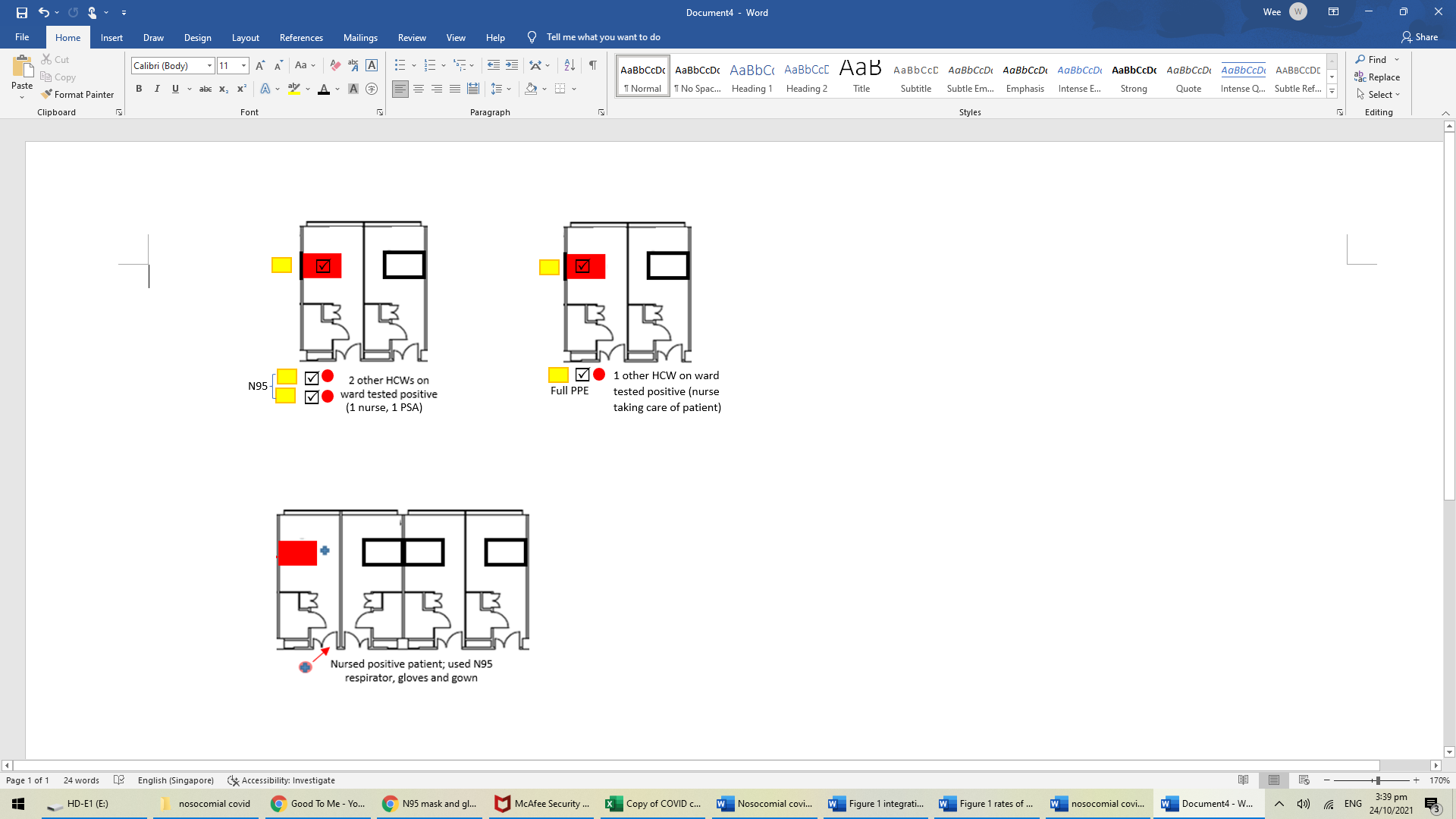 |
| Renal ward (cohort ward) | Inpatient-only (N=6) | 163 (fully vaccinated) | 32 (21 fully vaccinated, 11 partially/ unvaccinated) | No | Yes, epi cluster 3. 6 inpatient cases on the same ward | Yes but of the 6 inpatients, one in genomic cluster 1, 4 in genomic cluster 2, 1 unlinked | 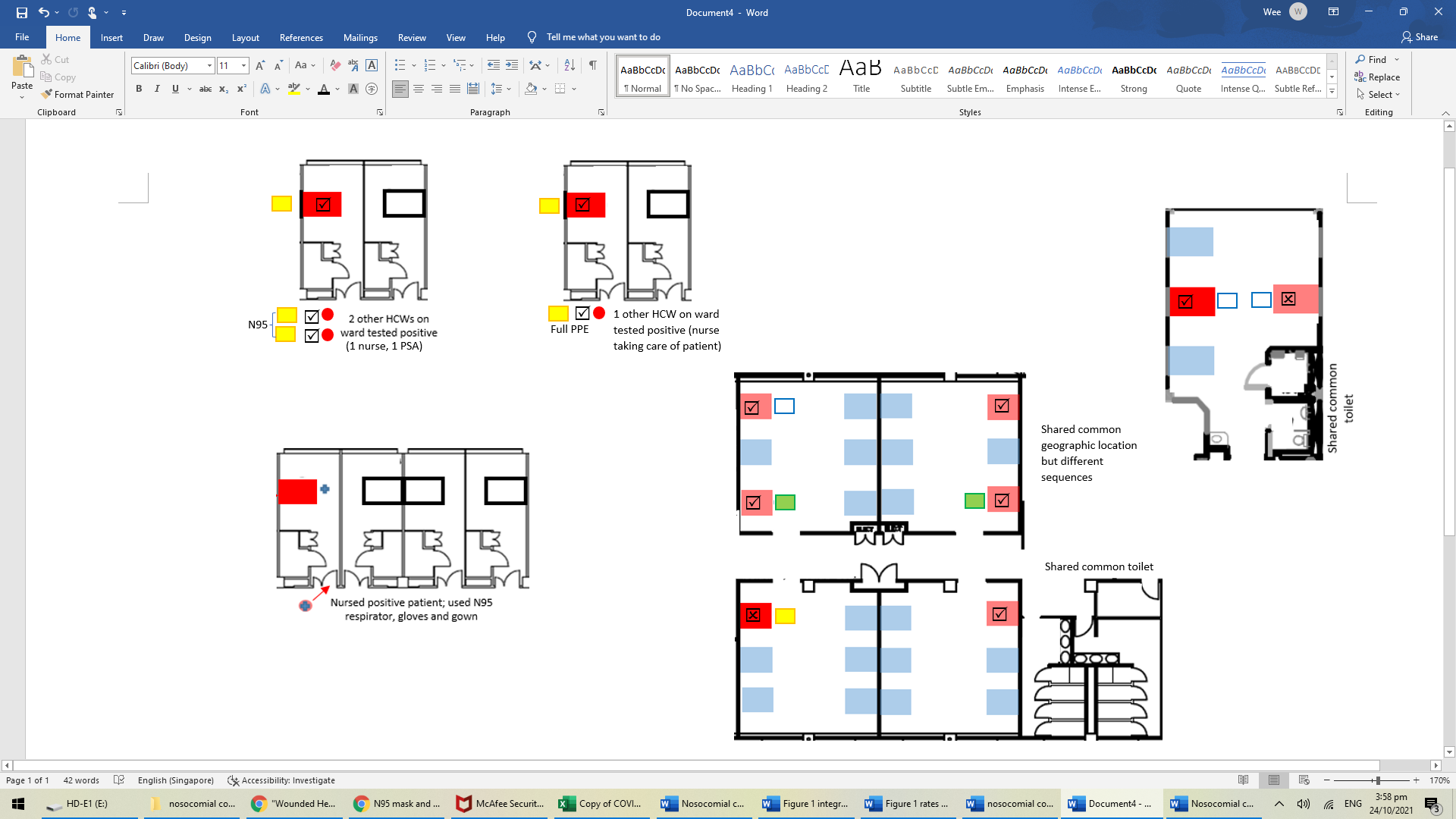 |
| Respiratory surveillance ward (enhanced precautions) | Inpatient-only (N=2) | 7 (fully vaccinated) | 3 (2 fully vaccinated, 1 partially/ unvaccinated) | Index identified as community-onset case | Yes, epi cluster 4. Case shared cubicle with community-onset C+ case and subsequently tested C+ within incubation period | **Genetic link could not be established. Unlinked sequences**; suggesting community acquisition in both cases | 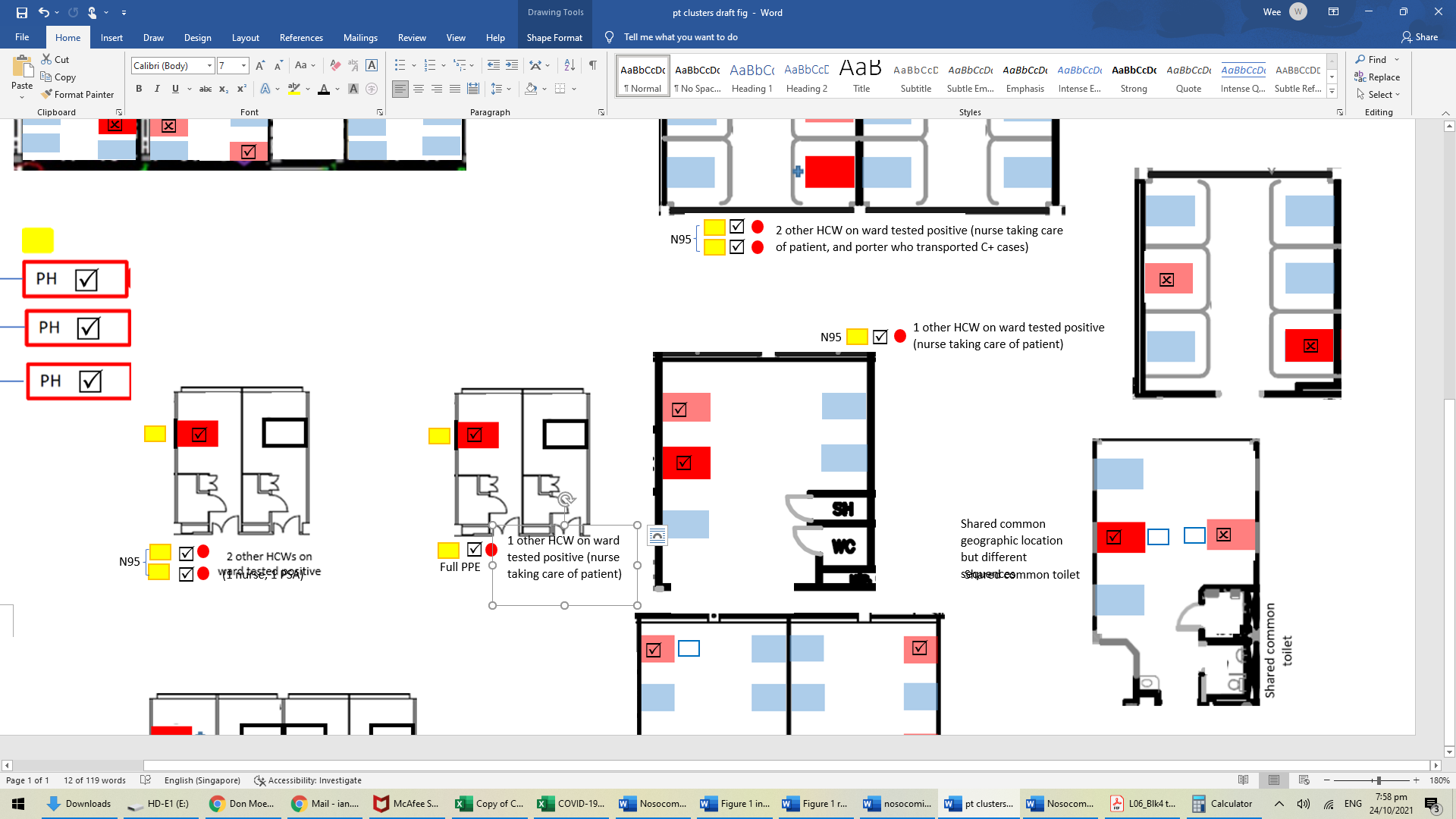 |
| General ward (cohort) | Mixed (N=13) | 249 (fully vaccinated) | 72 (58 fully vaccinated, 14 partially/ unvaccinated) | Index identified as community-onset case | Yes, epi cluster 5. Index was community-onset C+ case. 5 other inpatient cases on ward subsequently tested C+ within incubation period, together with 2 HCWs; 1 of the inpatient cases seeded secondary cases in other ward (3 inpatients, 2 staff) | Yes, genomic cluster 3. Secondary transmission was confirmed on sequencing as cases in different wards were linked. Of the 9 inpatient cases, 6 were linked, 3 could not be sequenced (low viral load); of the 4 HCW cases, 2 were linked, 1 could not be sequenced ( low viral load), 1 was unlinked. | 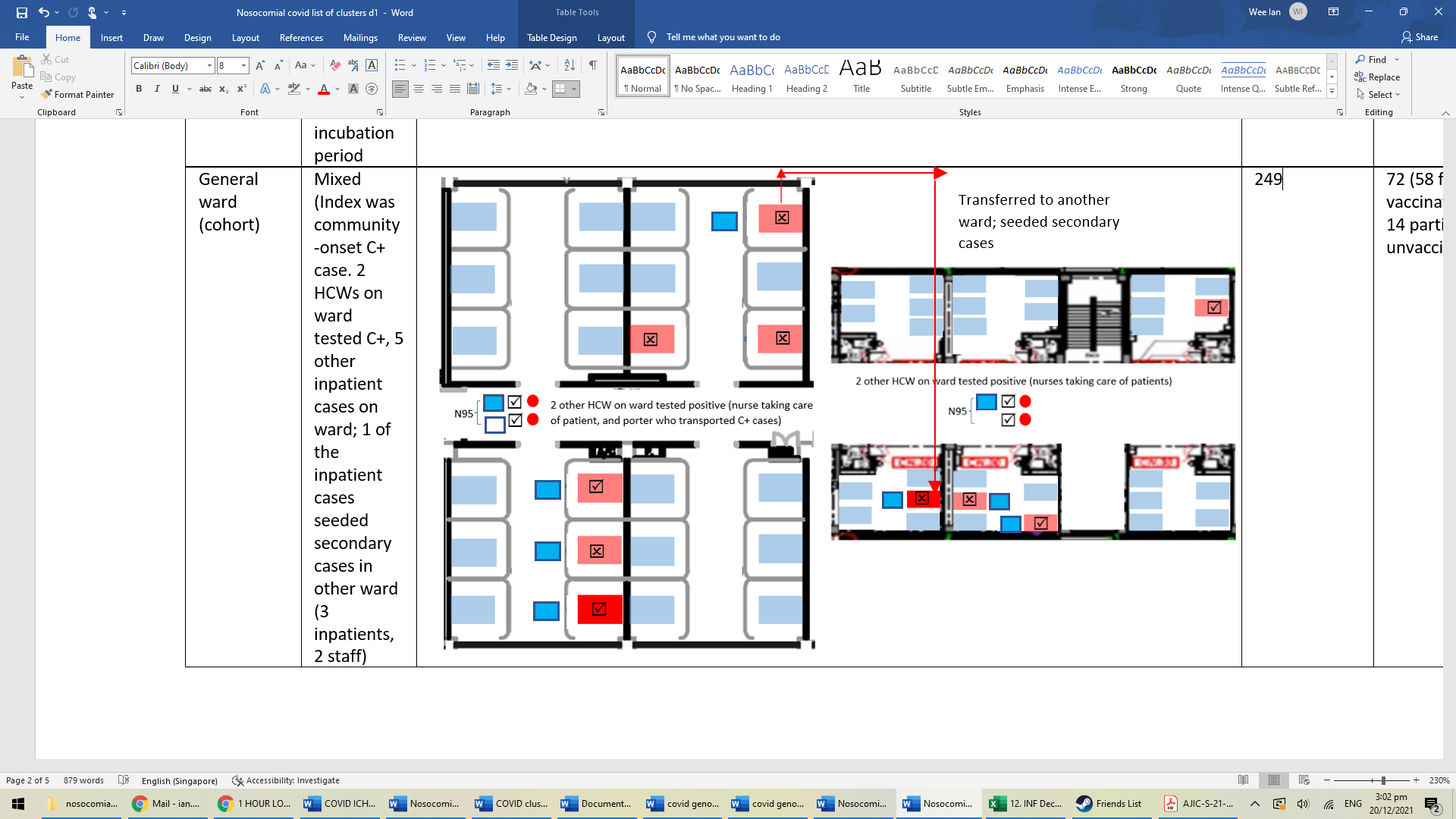 |
| General ward (cohort) | Mixed (N=5) | 42 (fully vaccinated) | 4 (2 fully vaccinated, 2 partially/ unvaccinated) | No | Part of epi cluster 6. 4 inpatient cases in same cubicle, 1 HCW in close-contact with the patients | Yes, genomic cluster 4. All five cases linked genetically. | 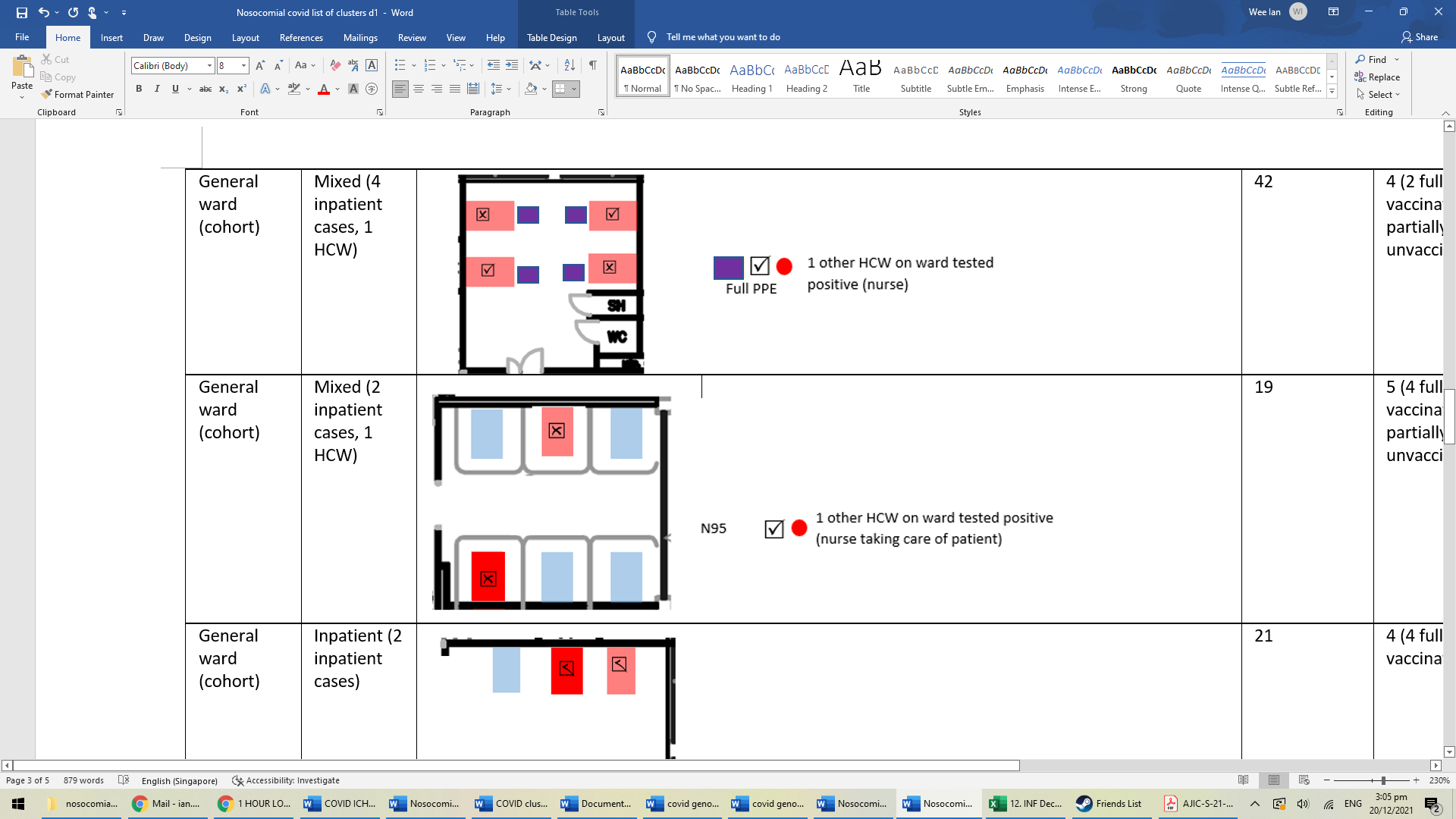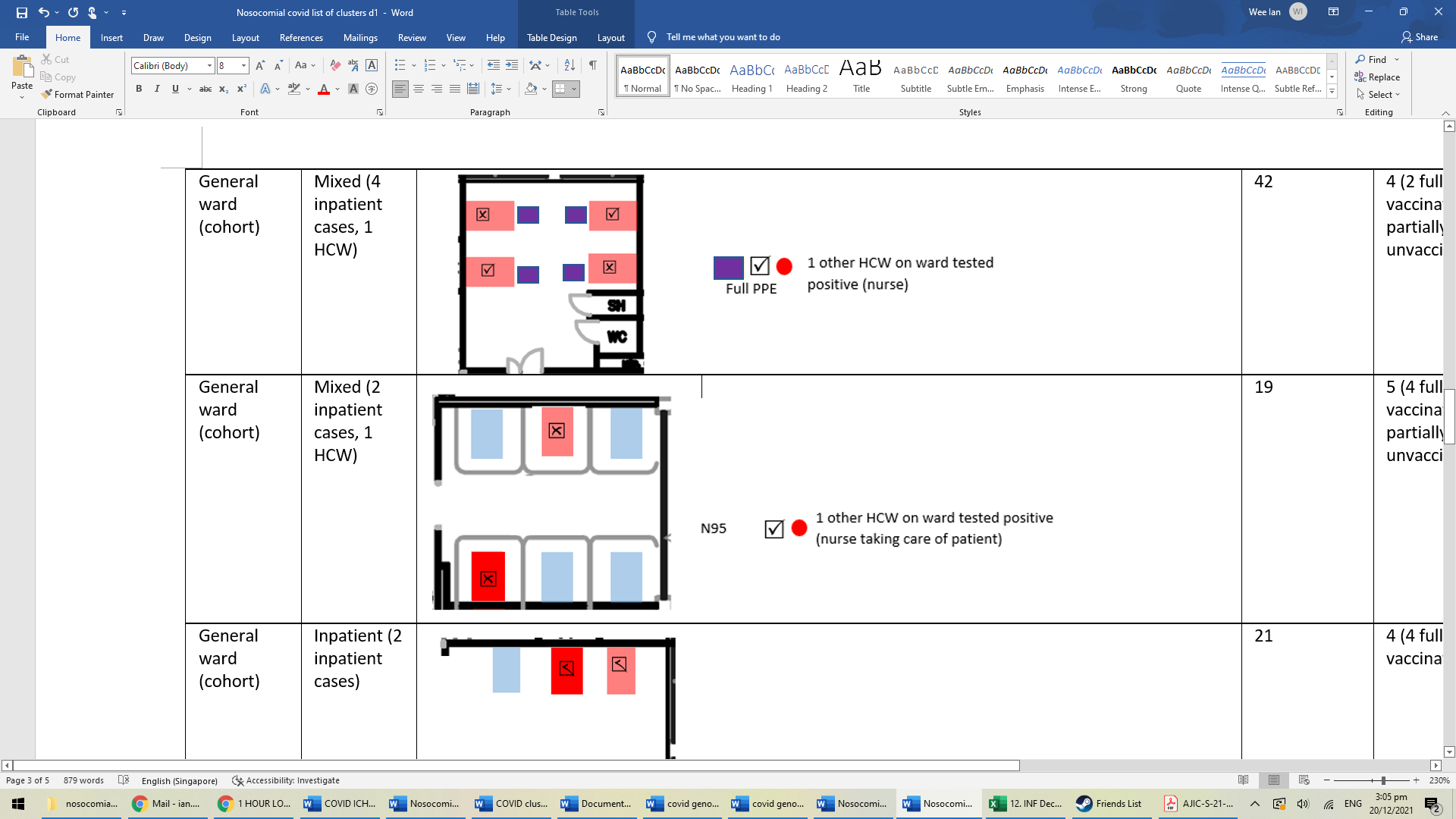 |
| General ward (cohort) | Mixed (N=3) | 19 (fully vaccinated) | 5 (4 fully vaccinated, 1 partially/ unvaccinated) | Index identified as indeterminate HA-onset case (tested positive on D3 of admission) | Part of epi cluster 7. 2 inpatient cases in same cubicle, 1 HCW in close-contact with the patients | **Genetic link could not be established.** Index case not linked to other HA-COVID-19 cases; other cases in cluster could not be sequenced (low viral load) | 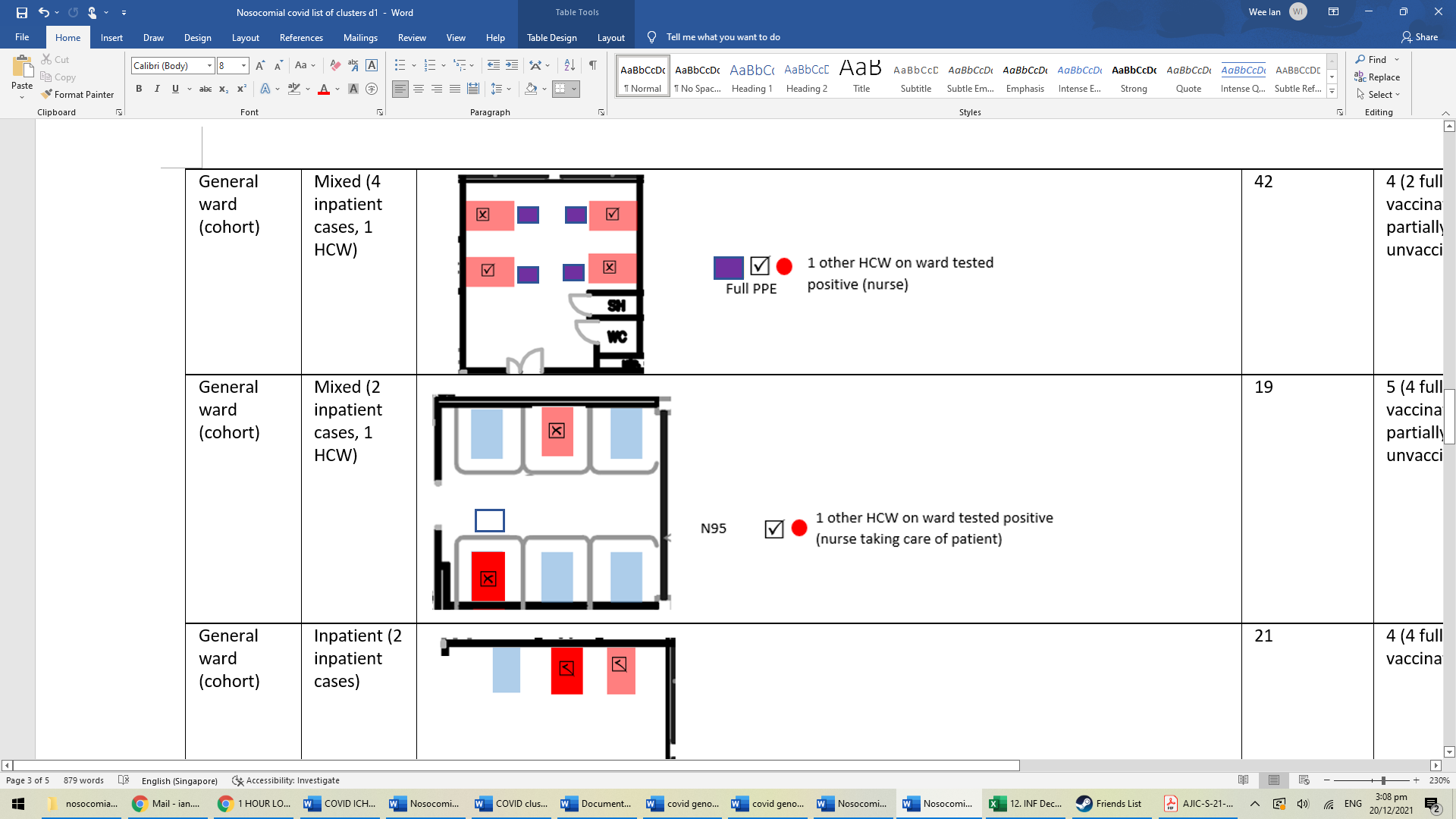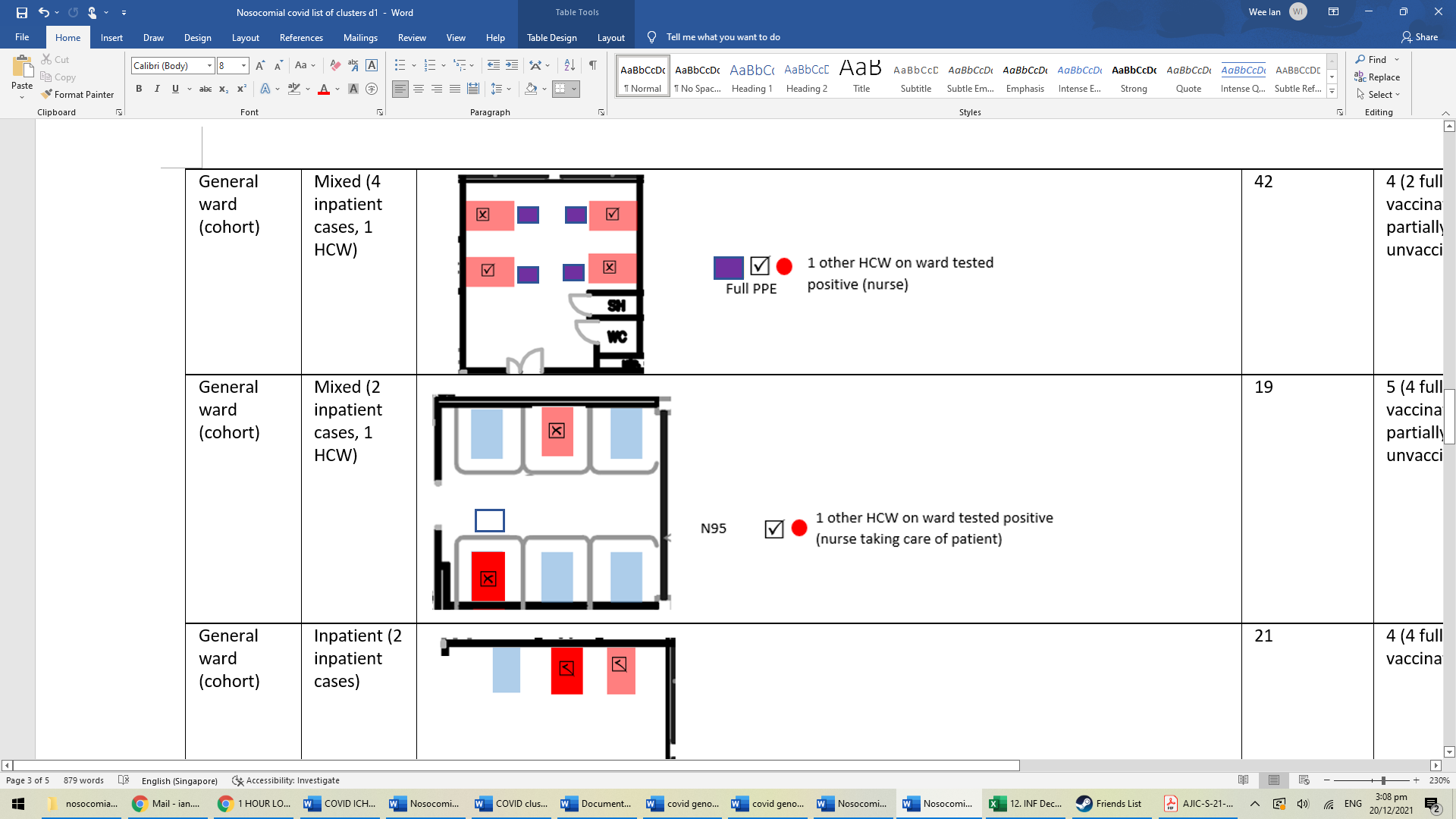 |
| General ward (cohort) | Inpatient-only (N=2) | 21(fully vaccinated) | 4 (4 fully vaccinated) | Index identified as indeterminate HA-onset case (tested positive on D3 of admission) | Part of epi cluster 8. 2 inpatient cases in same cubicle. | **Genetic link could not be established.** Index case not linked to other HA-COVID-19 cases; other cases in cluster could not be sequenced (low viral load) | 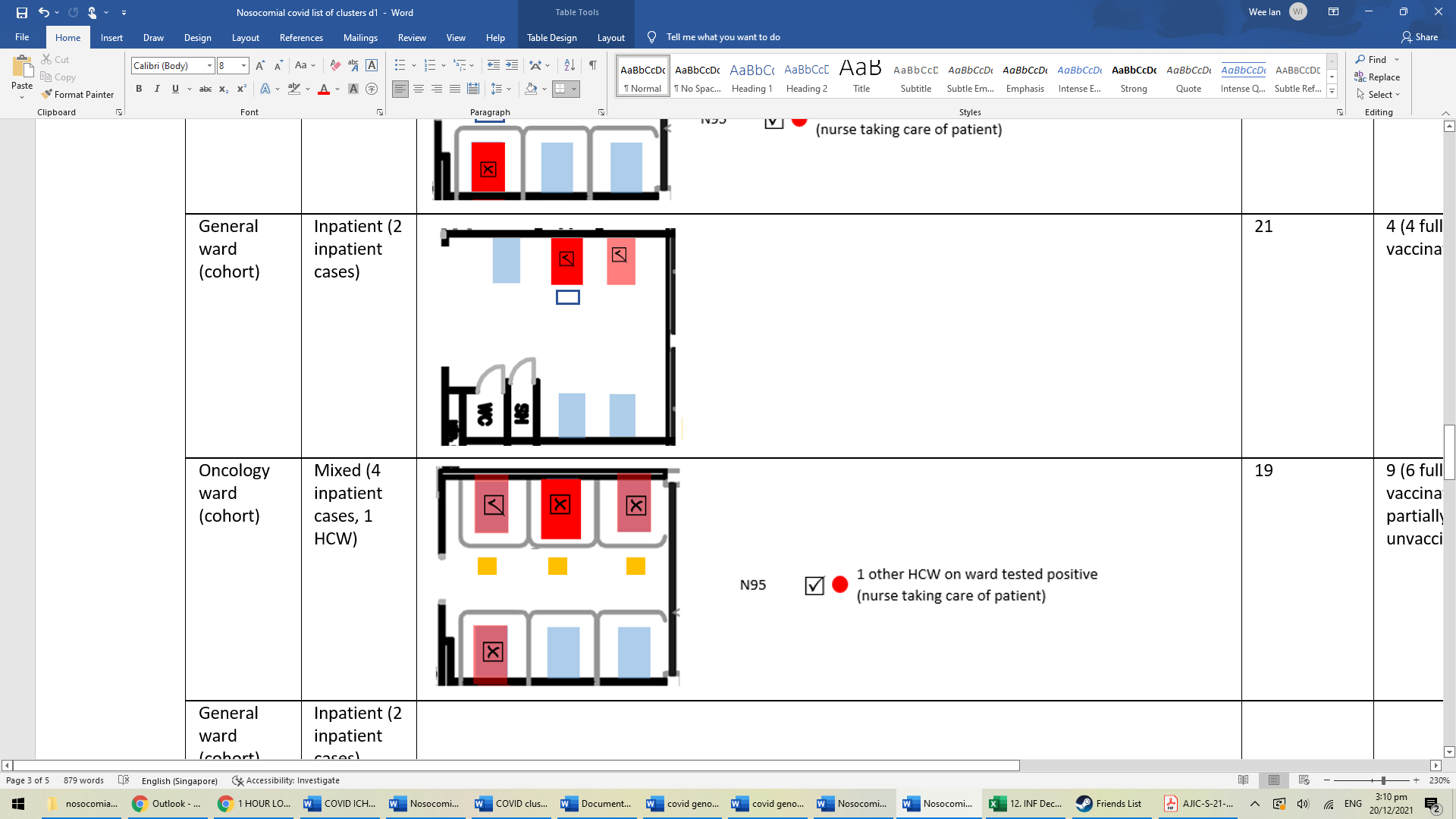 |
| Oncology ward (cohort) | Mixed (N=5) | 19 (fully vaccinated) | 9 (6 fully vaccinated, 3 partially/ unvaccinated) | Index identified as indeterminate HA-onset case (tested positive on D3 of admission) | Part of epi cluster 9. 4 inpatient cases in same cubicle, 1 HCW in close-contact with the patients | Yes. Genomic cluster 5. Of the 4 inpatient cases, 3 were linked genetically; remaining inpatient case and HCW case could not be sequenced (low viral load) | 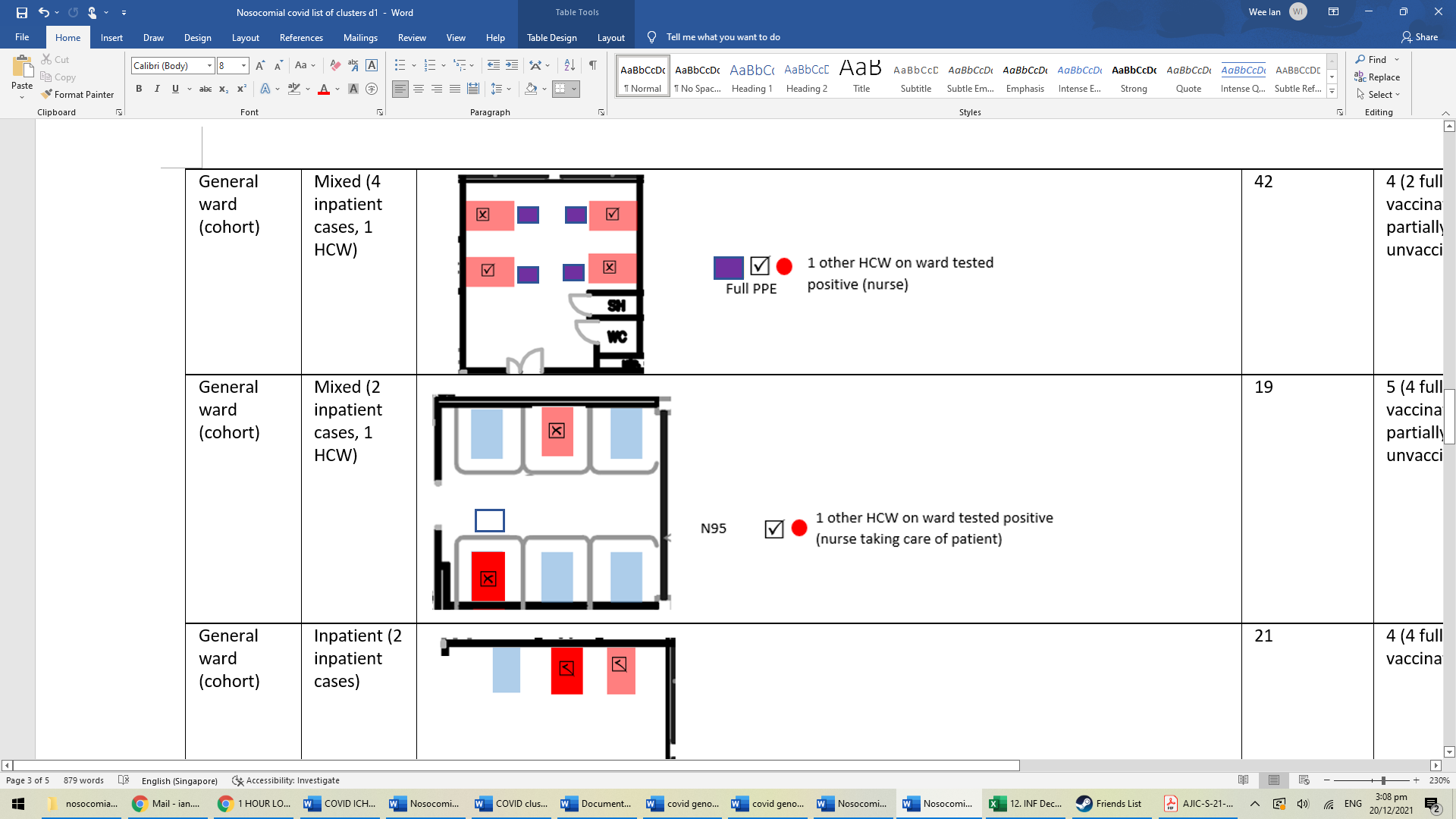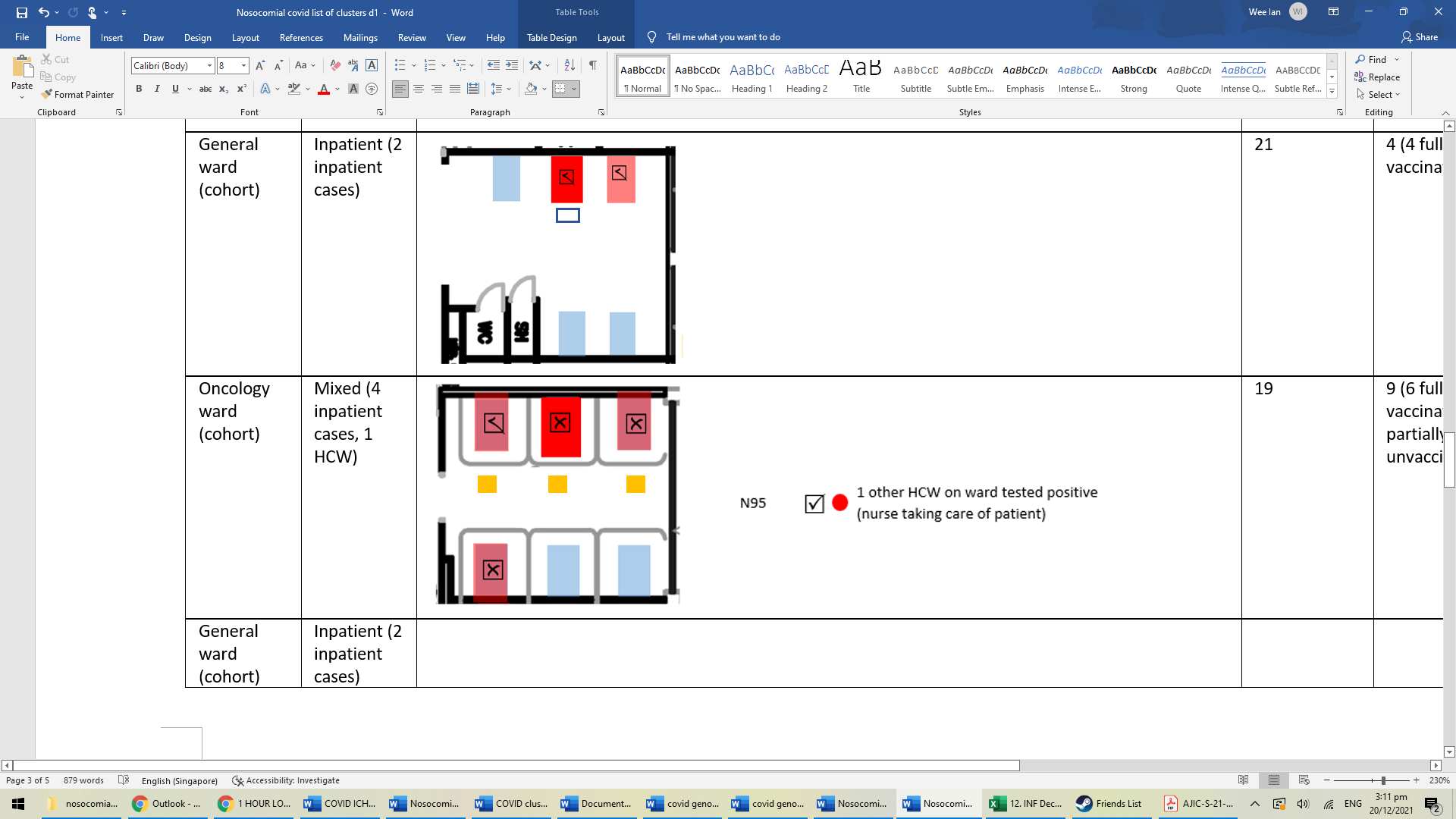 |
| General ward (cohort) | Inpatient-only (N=3) | 10 (fully vaccinated) | 5 (4 fully vaccinated, 1 partially/unvaccinated) | Index identified as indeterminate HA-onset case (tested positive on D4 of admission; household contact of index tested positive on D3) | Part of epi cluster 10. 2 inpatient cases in same cubicle. | Yes. Genomic cluster 6. 2 cases linked genetically; remaining inpatient case could not be sequenced (low viral load) | 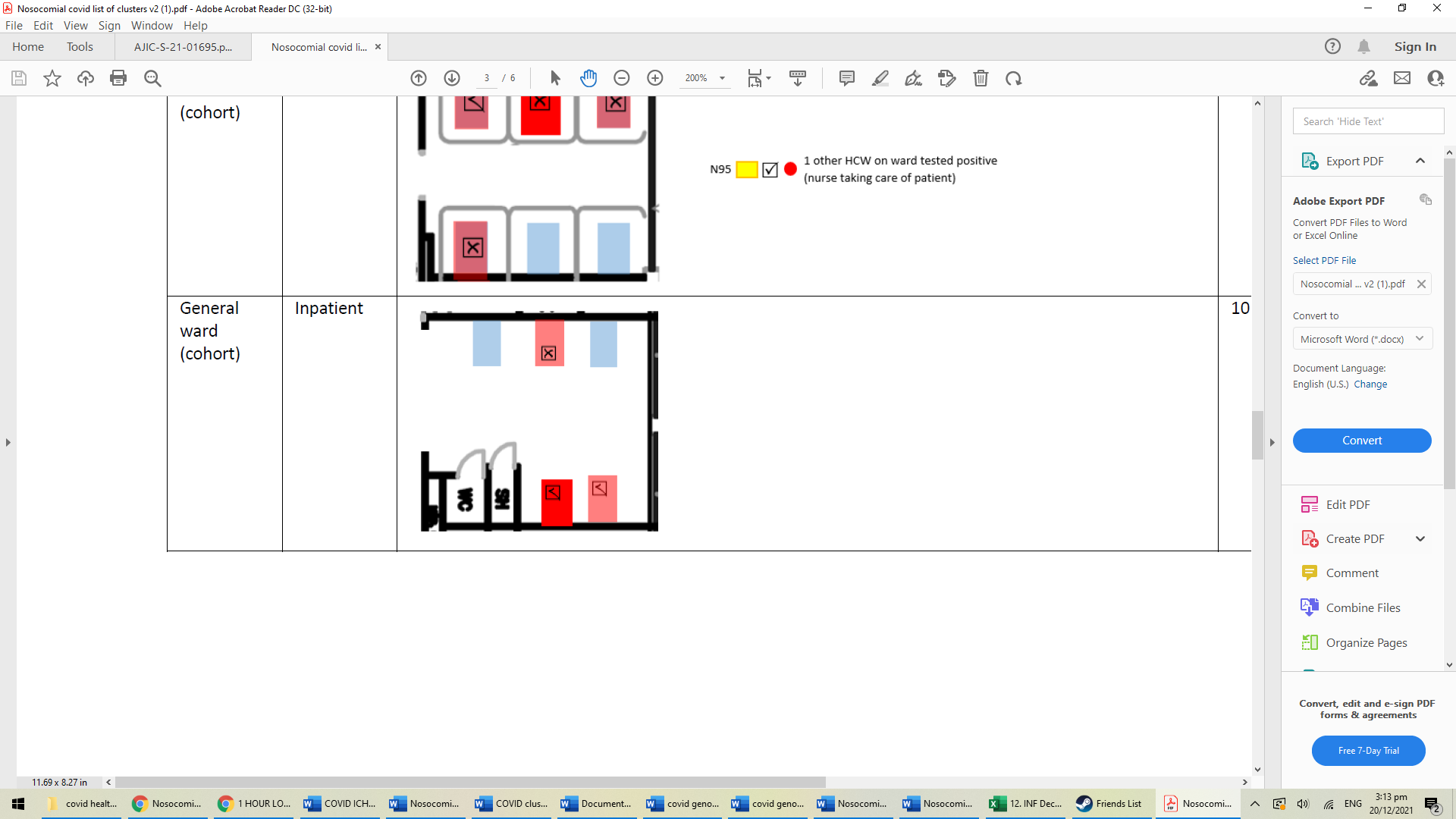 |
| General ward (cohort) | Inpatient-only (N=2) | 8 (fully vaccinated) | 2 (1 fully vaccinated, 1 partially/unvaccinated) | Index identified as community-onset case | Yes, epi cluster 11. Case shared cubicle with community-onset C+ case and subsequently tested C+ within incubation period | **Genetic link could not be established.** Index case not linked to other HA-COVID-19 cases; other cases in cluster could not be sequenced (low viral load) | 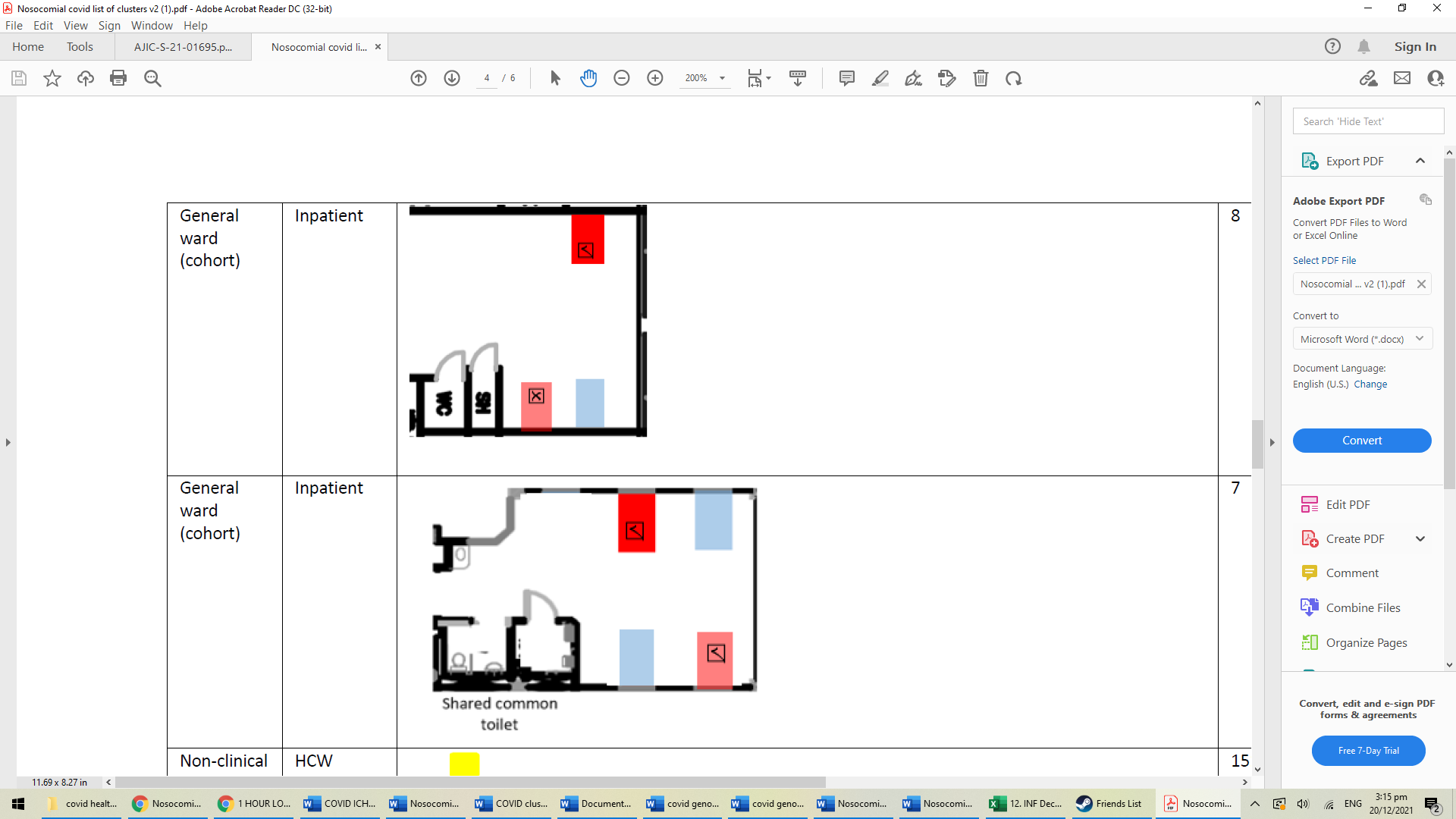 |
| General ward (cohort) | Inpatient-only (N=2) | 7 (fully vaccinated) | 3 (3 fully vaccinated) | Index identified as community-onset case | Yes, epi cluster 12. Case shared cubicle with community-onset C+ case and subsequently tested C+ within incubation period | **Genetic link could not be established.** Index case not linked to other HA-COVID-19 cases; other cases in cluster could not be sequenced (low viral load) | 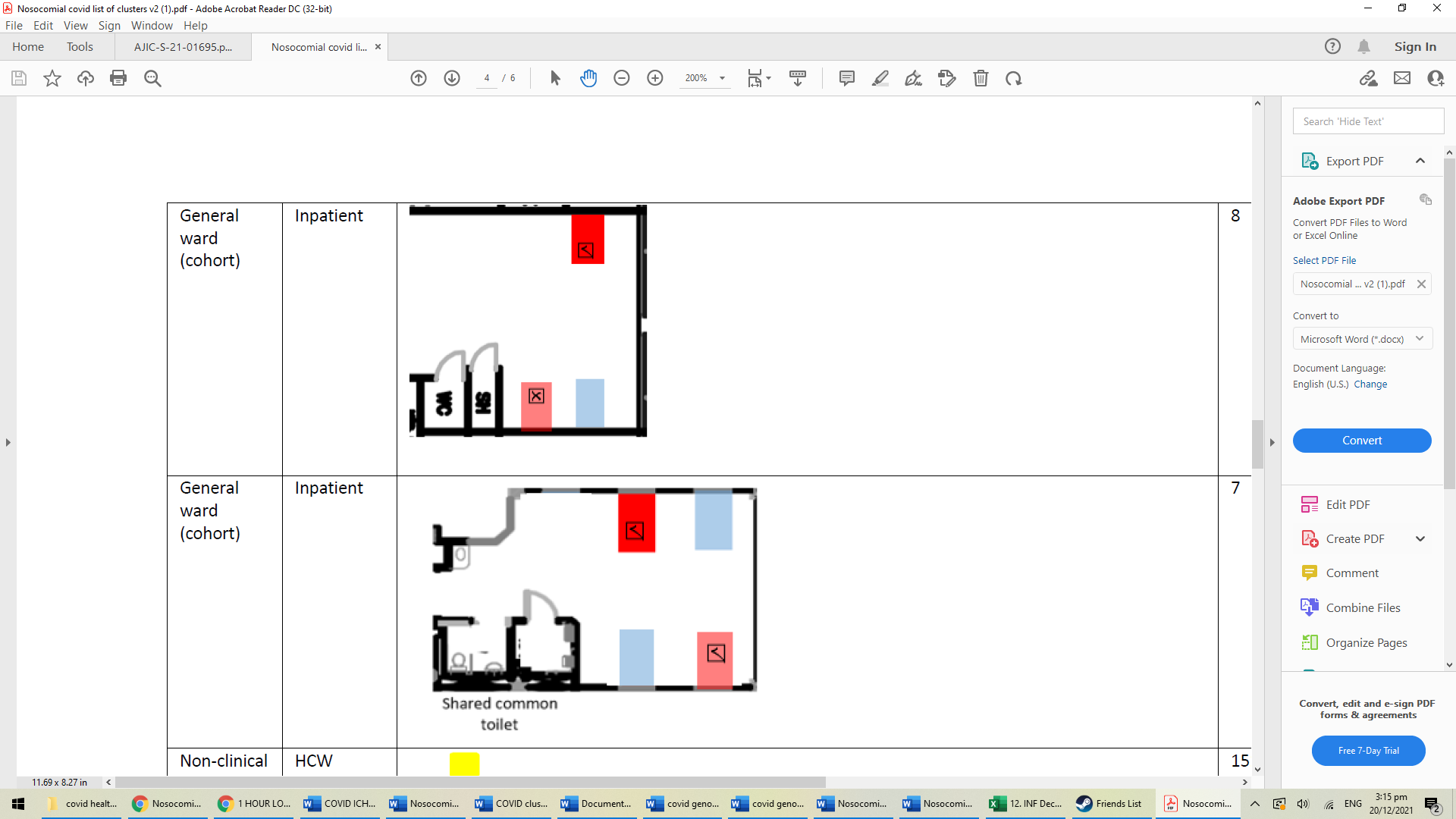 |
| **Clusters of potential healthcare-associated COVID-19 infection involving only HCW cases on initial epidemiological investigation** | | | | | | |  |
| Non-clinical area (inpatient pharmacy) | HCW (ancillary) (N=3) | 151 (all HCWs in inpatient pharmacy screened in view of cluster) | 0 | No | Part of HCW cluster 1; 3 inpatient pharmacy staff tested C+ | Yes, 1/3 HCWs in cluster linked to other cases in genomic cluster 1 | 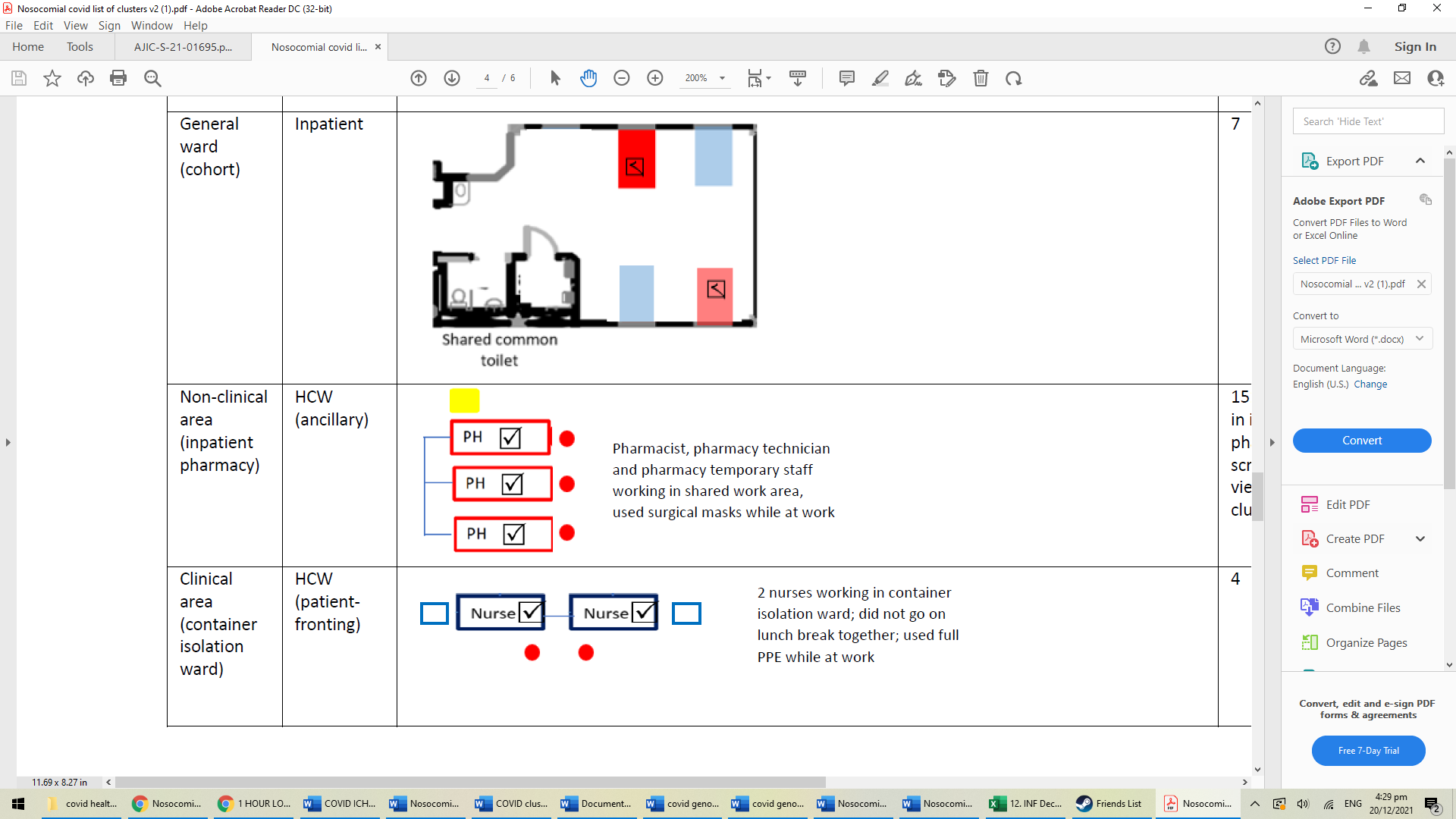 |
| Clinical area (container isolation ward) | HCW (patient-fronting) (N=2) | 4 | 0 | No | Part of HCW cluster 2; 2 nurses from container isolation ward tested C+ | **Genetic link could not be established. Unlinked sequences**; suggesting community acquisition in both cases | 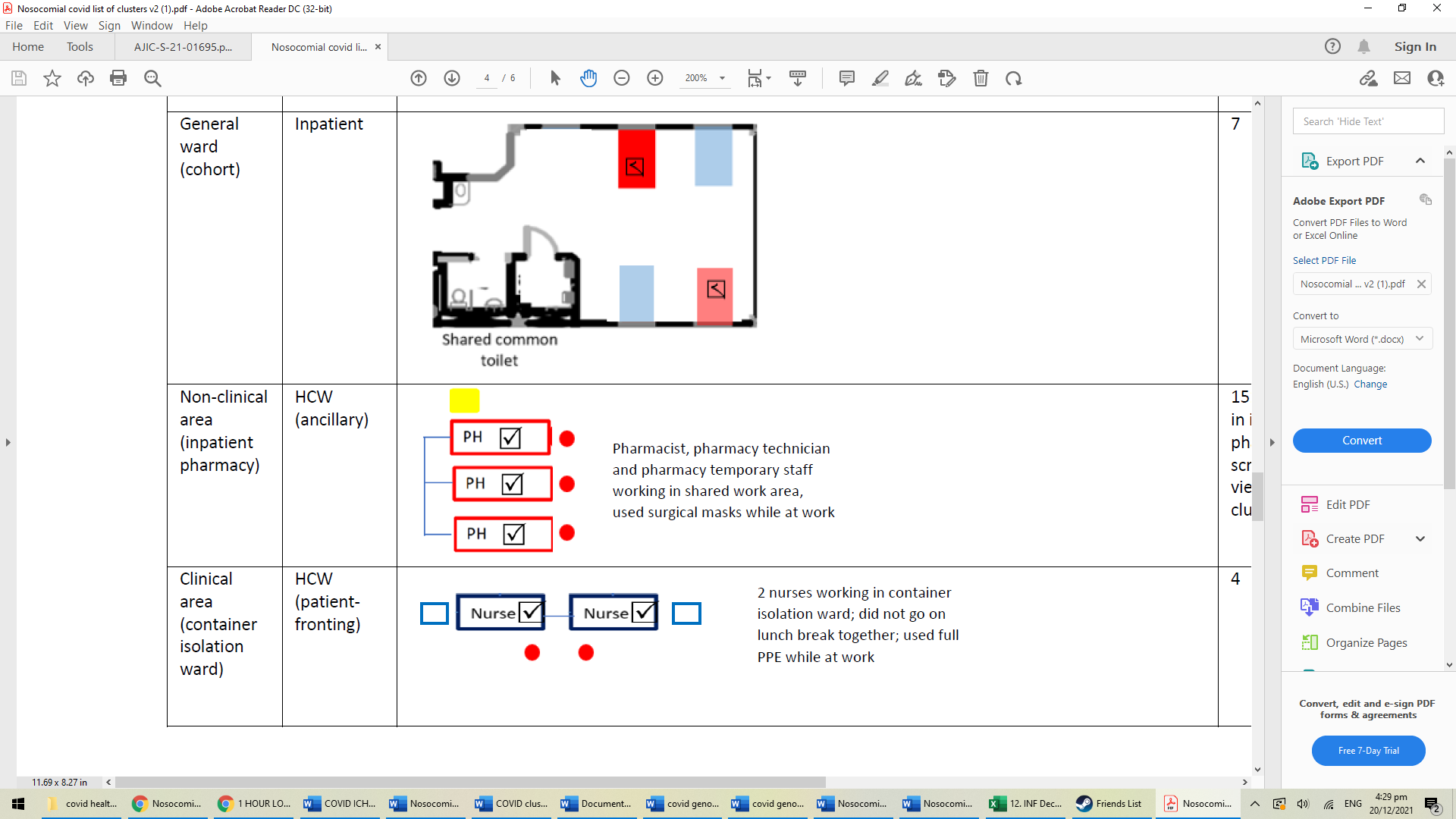 |
| Non-clinical area (inpatient pharmacy) | HCW (ancillary) (N=6) | 45 | 0 | One of the porters had visited a known community cluster of C+ cases | Part of HCW cluster 3; 6 porters linked to inpatient pharmacy tested C+ | Yes, 1/7 HCWs in cluster linked to other cases in genomic cluster 1; 1/7 HCW in cluster linked to genomic cluster 2 | 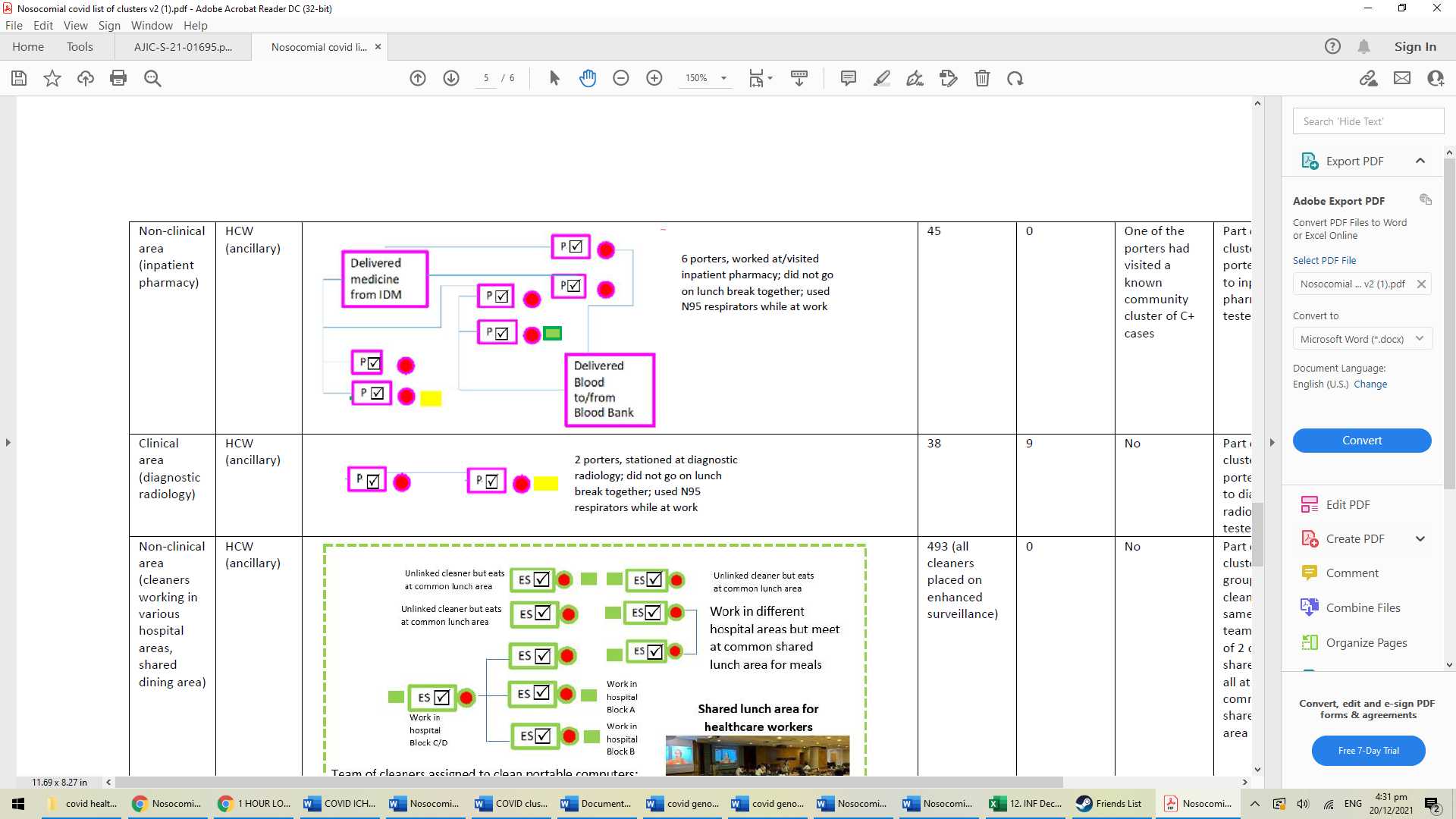 |
| Clinical area (diagnostic radiology) | HCW (ancillary) (N=2) | 38 | 9 | No | Part of HCW cluster 4; 2 porters linked to diagnostic radiology tested C+ | Yes, 1/2 HCWs in cluster linked to other cases in genomic cluster 1 | 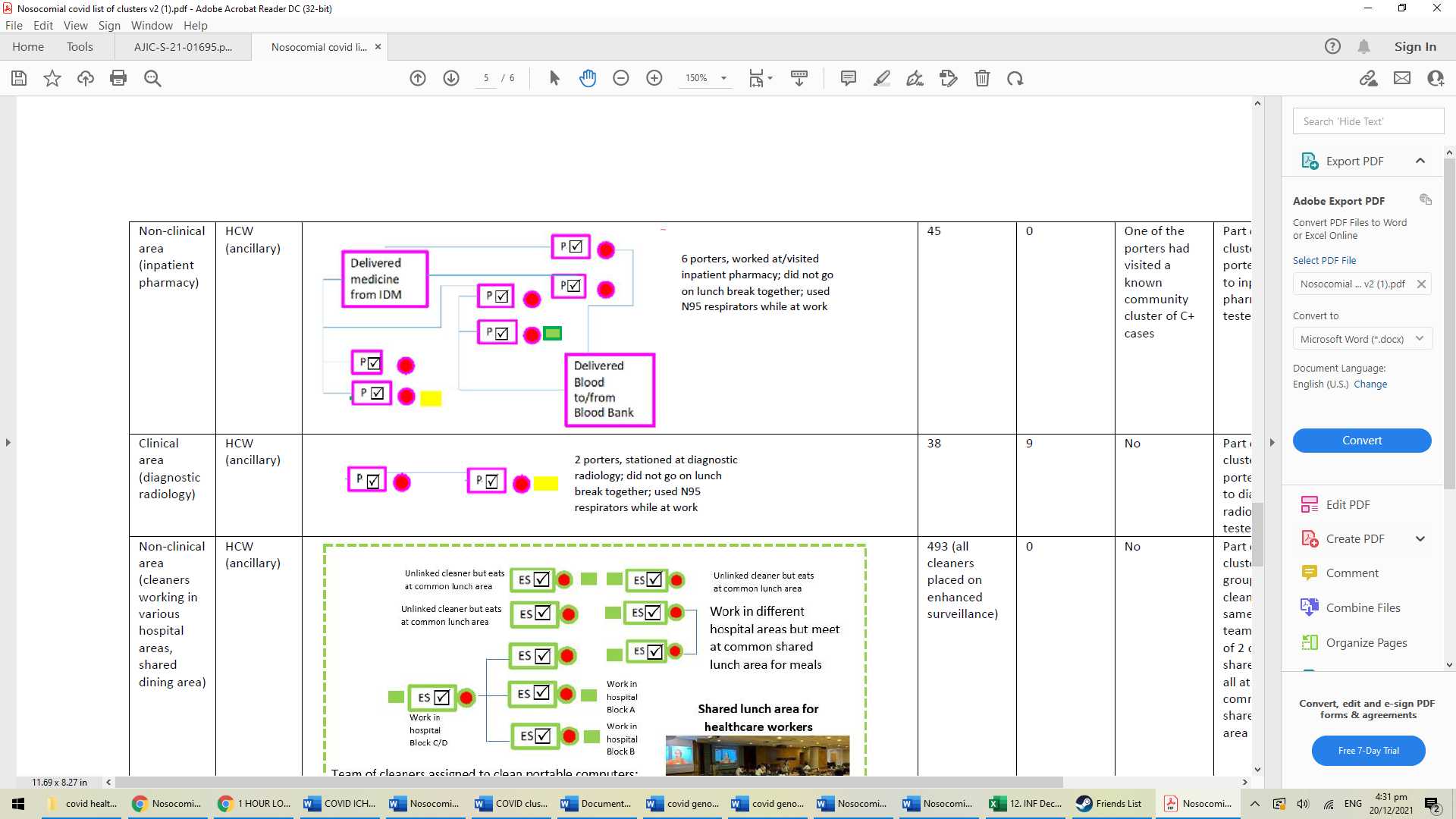 |
| Non-clinical area (cleaners working in various hospital areas, shared dining area) | HCW (ancillary) (N=9) | 493 (all cleaners placed on enhanced surveillance) | 0 | No | Part of HCW cluster 5; 1 group of 4 cleaners in same work team; 1 group of 2 cleaners shared lunch; all ate in common shared lunch area | 7/9 HCWs in cluster linked to other cases in genomic cluster 2 | 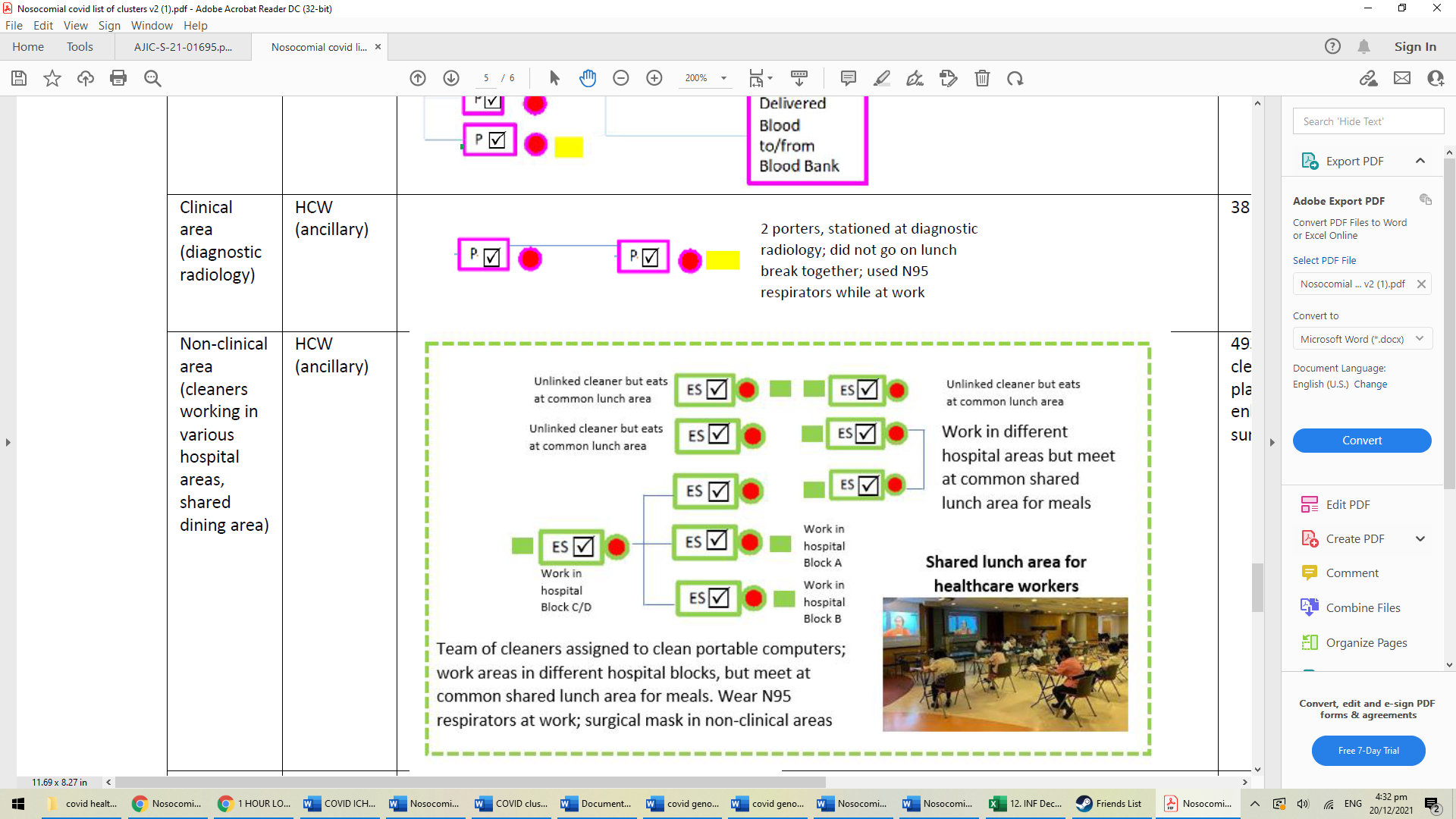 |
| Non-clinical area (security office) | HCW (ancillary) (N=2) | 81 | 0 | No | Part of HCW cluster 6; 2 security officers sharing an office tested C+ | **Genetic link could not be established.** Index case not linked to other HA-COVID-19 cases; other cases in cluster could not be sequenced (low viral load) | 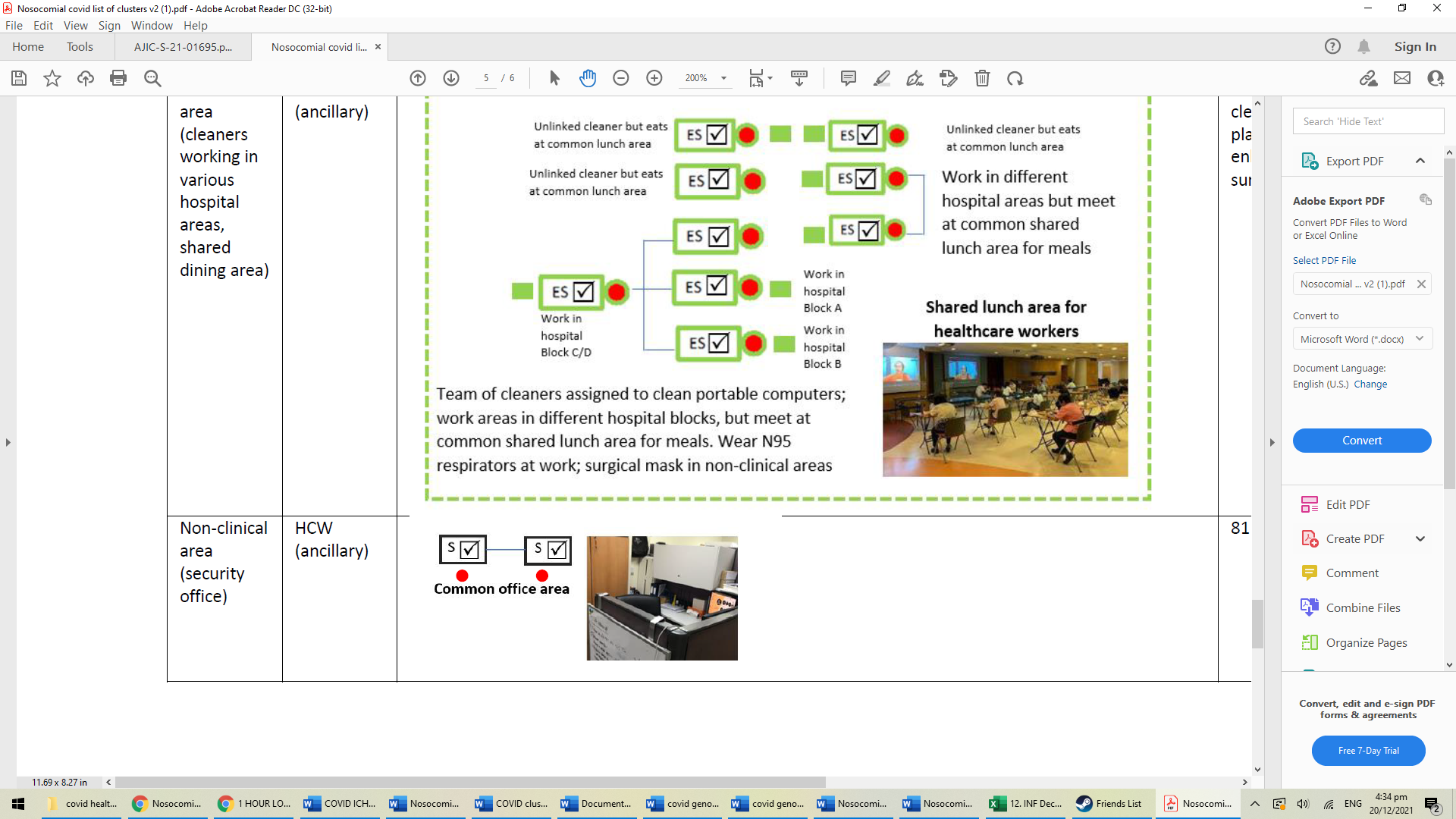 |
| Non-clinical area (workshop) | HCW (ancillary) (N=2) | 3 | 0 | No | Part of HCW cluster 7; 2 maintenance technicians tested C+ | **Genetic link could not be established. Unlinked sequences**; suggesting community acquisition in both cases | 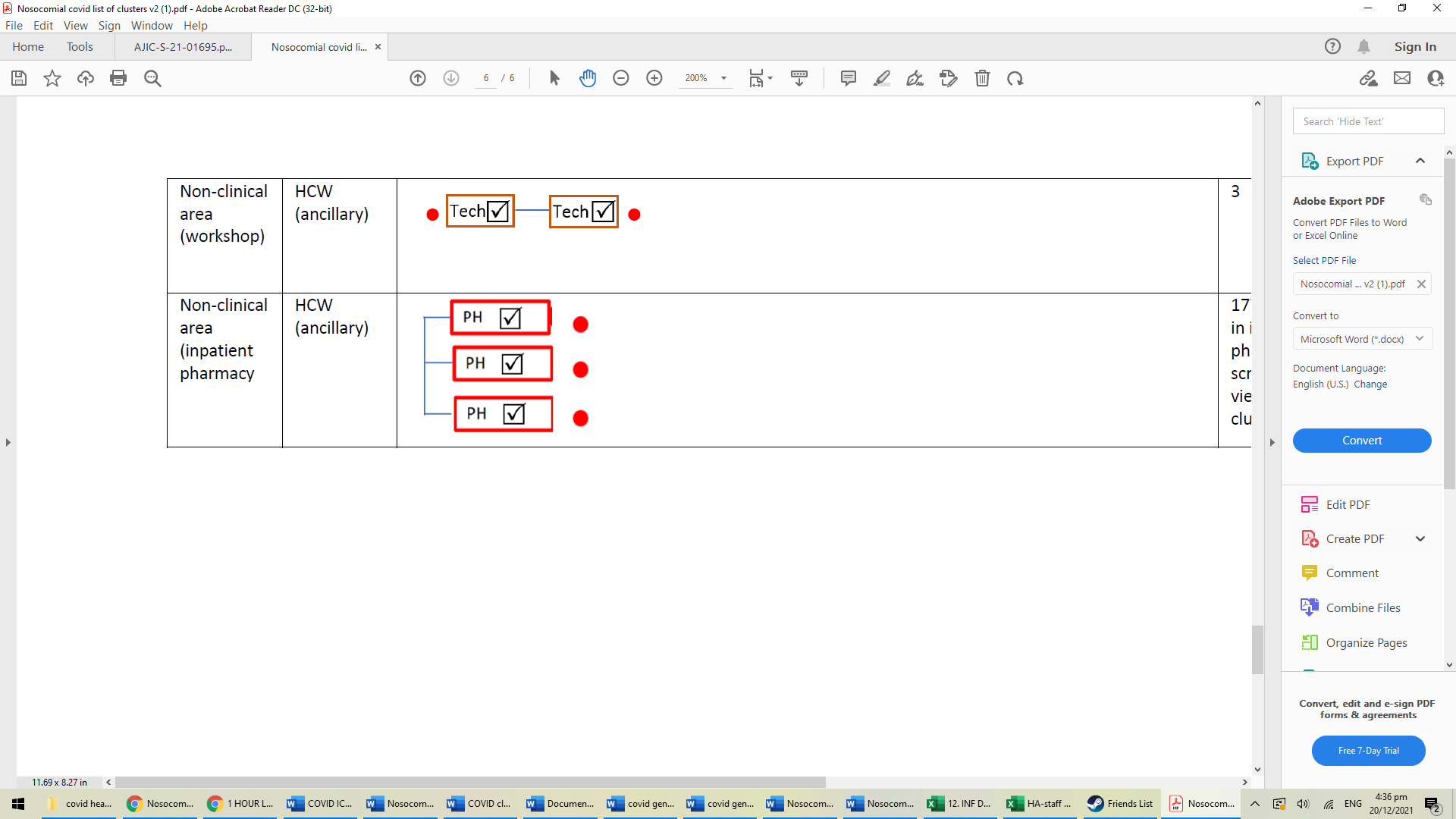 |
| Non-clinical area (inpatient pharmacy | HCW (ancillary) (N=3) | 177 (all HCWs in inpatient pharmacy screened in view of cluster) | 0 | No | Part of HCW cluster 8; 3 inpatient pharmacy staff tested C+ | **Genetic link could not be established.** Index case not linked to other HA-COVID-19 cases; other cases in cluster could not be sequenced (low viral load) | 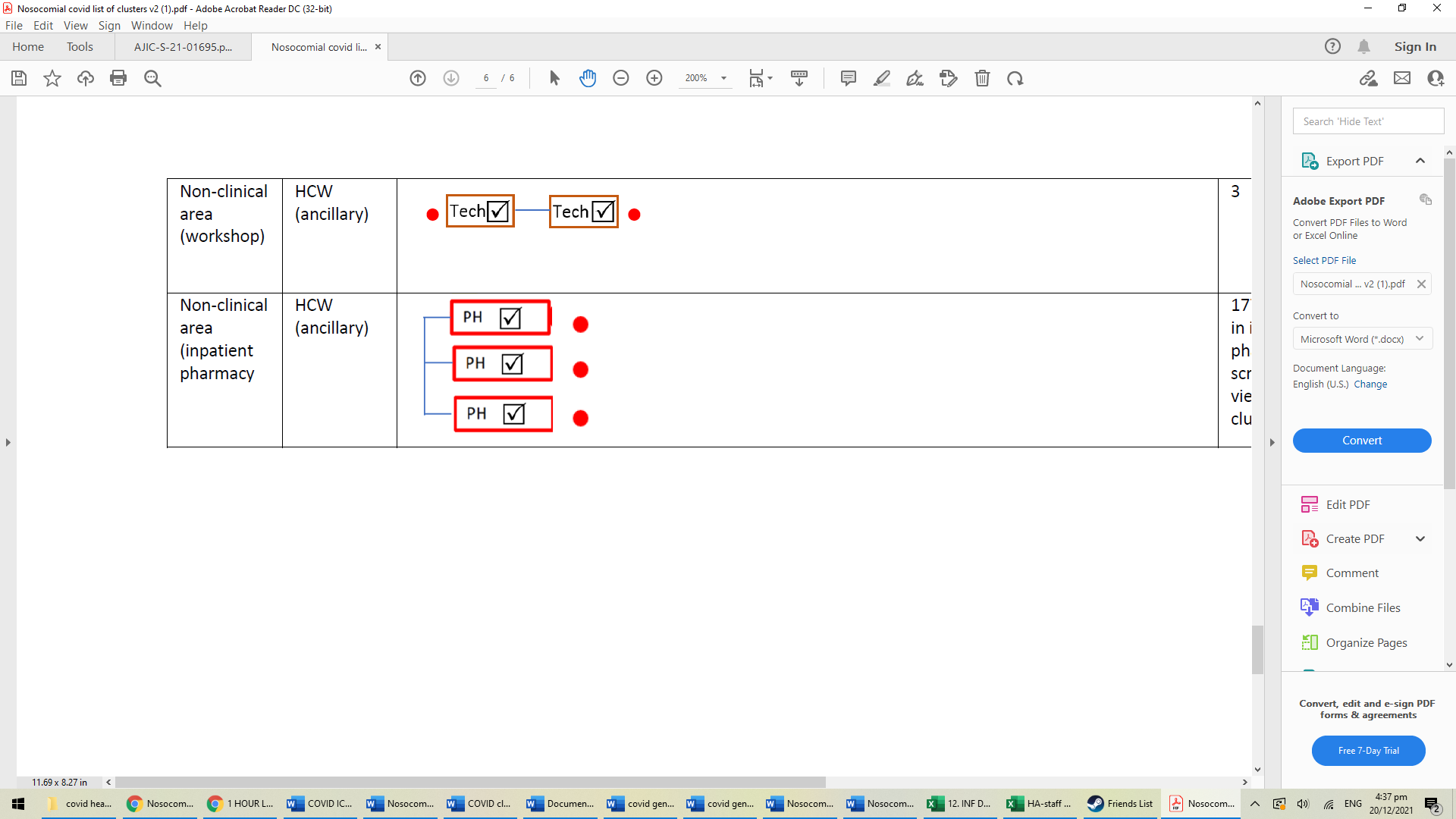 |
