# Supplementary material for: Linking sporadic hospital clusters during a community surge of the severe acute respiratory coronavirus virus 2 (SARS-CoV-2) B.1.617.2 delta variant: The utility of whole-genome sequencing
Source: Infect Control Hosp Epidemiol. 2022 Apr 27;44(6):1014–8. doi: 10.1017/ice.2022.106 (PMC10262163; doi:10.1017/ice.2022.106)
Supplement: Supplementary file 1 [file S0899823X22001064sup.zip › S0899823X22001064sup002.docx]

**Supplementary Table 1: Potential healthcare-associated COVID-19 inpatient cases (N=45) within a Singaporean tertiary hospital, over a 5-month period of enhanced surveillance**

| **Inpatient case number** | **Classification (indeterminate, probable, definite HA-COVID-19)** | **Vaccination status** | **Plausible source of COVID-19 infection based on epidemiological investigations alone** | **Plausible source of COVID-19 infection based on epidemiological investigations and whole-genome-sequencing** |
| --- | --- | --- | --- | --- |
| 1 | Definite | Unvaccinated | Had C+ visitor; no epidemiological link to other inpatient or HCW COVID-19 cases; plausible transmission from C+ visitor | Linked to other inpatient/HCW COVID-19 cases on sequencing (genomic cluster 1); transmission from C+ visitor deemed less plausible |
| 2 | Probable | Fully vaccinated | 2 HCWs on ward tested C+; likely intra-ward transmission | Linked to other inpatient/HCW COVID-19 cases on sequencing (genomic cluster 1); transmission beyond ward plausible |
| 3 | Indeterminate | Unvaccinated | Had C+ visitor; no epidemiological link to other inpatient or HCW COVID-19 cases | No epidemiological or sequencing link to other inpatient or HCW COVID-19 cases; **reclassified as** **plausible community-onset-case** |
| 4 | Indeterminate | Unvaccinated | No epidemiological link to other inpatient or HCW COVID-19 cases | No epidemiological or sequencing link to other inpatient or HCW COVID-19 cases; **reclassified as** **plausible community-onset-case** |
| 5 | Definite | Fully vaccinated | Had C+ visitor. 1 HCW on ward tested C+; likely intra-ward transmission | Linked to other inpatient/HCW COVID-19 cases on sequencing (genomic cluster 1); transmission beyond ward plausible |
| 6 | Probable | Unvaccinated | 6 inpatient cases on ward; likely intra-ward transmission | Linked to other inpatient/HCW COVID-19 cases on sequencing (genomic cluster 1); transmission beyond ward plausible. **Not linked to other inpatient cases on ward.** |
| 7 | Probable | Fully vaccinated | 6 inpatient cases on ward; likely intra-ward transmission | Linked to other inpatient/HCW COVID-19 cases on sequencing (genomic cluster 2); transmission beyond ward plausible |
| 8 | Probable | Fully vaccinated | 6 inpatient cases on ward; likely intra-ward transmission | Linked to other inpatient/HCW COVID-19 cases on sequencing (genomic cluster 2); transmission beyond ward plausible |
| 9 | Probable | Fully vaccinated | 6 inpatient cases on ward; likely intra-ward transmission | Linked to other inpatient/HCW COVID-19 cases on sequencing (genomic cluster 2); transmission beyond ward plausible |
| 10 | Probable | Fully vaccinated | 6 inpatient cases on ward; likely intra-ward transmission | No sequencing link to other inpatient or HCW COVID-19 cases;  **reclassified as** **plausible community-onset case** (was discharged post-exposure and readmitted) |
| 11 | Probable | Fully vaccinated | 6 inpatient cases on ward; likely intra-ward transmission | Linked to other inpatient/HCW COVID-19 cases on sequencing (genomic cluster 2); transmission beyond ward plausible |
| 12 | Probable | Partially vaccinated | Shared cubicle with community-onset C+ case; likely intra-ward transmission | No sequencing link to other inpatient or HCW COVID-19 cases;  **reclassified as plausible community-onset case** (was discharged post-exposure and readmitted) |
| 13 | Probable | Unvaccinated | Shared cubicle with community-onset C+ case. 2 HCW on ward tested C+, 5 inpatient cases on ward; likely intra-ward transmission | Linked to other inpatient/HCW COVID-19 cases on sequencing (genomic cluster 3); concordance between epidemiological and sequencing data |
| 14 | Probable | Unvaccinated | 2 HCW on ward tested C+, 5 inpatient cases on ward; likely intra-ward transmission | Sequencing not performed due to low viral load |
| 15 | Definite | Unvaccinated | 2 HCW on ward tested C+, 5 inpatient cases on ward; likely intra-ward transmission. Was subsequently transferred to another ward and seeded secondary cases in other ward (3 inpatients, 2 staff). | Linked to other inpatient/HCW COVID-19 cases on sequencing (genomic cluster 3); concordance between epidemiological and sequencing data |
| 16 | Probable | Fully vaccinated | 2 HCW on ward tested C+, 5 inpatient cases on ward; likely intra-ward transmission | Linked to other inpatient/HCW COVID-19 cases on sequencing (genomic cluster 3); concordance between epidemiological and sequencing data |
| 17 | Probable | Partially vaccinated | 2 HCW on ward tested C+, 5 inpatient cases on ward; likely intra-ward transmission | Sequencing not performed due to low viral load |
| 18 | Definite | Fully vaccinated | 2 HCW on ward tested C+; 3 inpatient cases on ward; likely intra-ward transmission (secondary cases seeded from initial cluster) | Linked to other inpatient/HCW COVID-19 cases on sequencing (genomic cluster 3); concordance between epidemiological and sequencing data |
| 19 | Definite | Unvaccinated | 2 HCW on ward tested C+; 3 inpatient cases on ward; likely intra-ward transmission (secondary cases seeded from initial cluster) | Linked to other inpatient/HCW COVID-19 cases on sequencing (genomic cluster 3); concordance between epidemiological and sequencing data 3 |
| 20 | Definite | Fully vaccinated | 2 HCW on ward tested C+; 3 inpatient cases on ward; likely intra-ward transmission (secondary cases seeded from initial cluster) | Linked to other inpatient/HCW COVID-19 cases on sequencing (genomic cluster 3); concordance between epidemiological and sequencing data |
| 21 | Definite | Unvaccinated | Had frequent visitors as was on palliative care; none known to be C+. No epidemiological link to other inpatient or HCW COVID-19 cases. Plausible transmission from undiagnosed C+ visitor | Linked to other inpatient/HCW COVID-19 cases on sequencing (genomic cluster 3); transmission beyond ward plausible |
| 22 | Indeterminate | Unvaccinated | Had C+ visitor; household cases C+, no epidemiological link to other inpatient or HCW COVID-19 cases; plausible transmission from C+ visitor | No epidemiological or sequencing link to other inpatient or HCW COVID-19 cases; **reclassified as** **plausible community-onset-case** |
| 23 | Definite | Unvaccinated | 1 HCW on ward tested C+, 4 inpatient cases on ward; likely intra-ward transmission | Linked to other inpatient/HCW COVID-19 cases on sequencing (genomic cluster 4); concordance between epidemiological and sequencing data |
| 24 | Definite | Unvaccinated | 1 HCW on ward tested C+, 4 inpatient cases on ward; likely intra-ward transmission | Linked to other inpatient/HCW COVID-19 cases on sequencing (genomic cluster 4); concordance between epidemiological and sequencing data |
| 25 | Definite | Fully vaccinated | 1 HCW on ward tested C+, 4 inpatient cases on ward; likely intra-ward transmission | Linked to other inpatient/HCW COVID-19 cases on sequencing (genomic cluster 4); concordance between epidemiological and sequencing data |
| 26 | Definite | Fully vaccinated | 1 HCW on ward tested C+, 4 inpatient cases on ward; likely intra-ward transmission | Linked to other inpatient/HCW COVID-19 cases on sequencing (genomic cluster 4); concordance between epidemiological and sequencing data |
| 27 | Indeterminate | Partially vaccinated | 1 HCW on ward tested C+, 1 inpatient case on ward; likely intra-ward transmission | No sequencing link to other inpatient or HCW COVID-19 cases; **reclassified as** **plausible community-onset-case** |
| 28 | Definite | Partially vaccinated | 1 HCW on ward tested C+, 1 inpatient case on ward; likely intra-ward transmission | Sequencing not performed due to low viral load |
| 29 | Indeterminate | Unvaccinated | No epidemiological link to other inpatient or HCW COVID-19 cases | No epidemiological or sequencing link to other inpatient or HCW COVID-19 cases; **reclassified as** **plausible community-onset-case** |
| 30 | Indeterminate | Partially vaccinated | No epidemiological link to other inpatient or HCW COVID-19 cases | No epidemiological or sequencing link to other inpatient or HCW COVID-19 cases; **reclassified as** **plausible ommunity-onset-case** |
| 31 | Indeterminate | Fully vaccinated | 1 inpatient case on ward; likely intra-ward transmission | No sequencing link to other inpatient or HCW COVID-19 cases; **reclassified as** **plausible community-onset-case** |
| 32 | Definite | Fully vaccinated | 1 inpatient case on ward; likely intra-ward transmission | Sequencing not performed due to low viral load |
| 33 | Probable | Fully vaccinated | No epidemiological link to other inpatient or HCW COVID-19 cases | No epidemiological or sequencing link to other inpatient or HCW COVID-19 cases. |
| 34 | Indeterminate | Partially vaccinated | 1 HCW on ward tested C+, 4 inpatient cases on ward; likely intra-ward transmission | Linked to other inpatient/HCW COVID-19 cases on sequencing (genomic cluster 5); concordance between epidemiological and sequencing data |
| 35 | Probable | Fully vaccinated | 1 HCW on ward tested C+, 4 inpatient cases on ward; likely intra-ward transmission | Linked to other inpatient/HCW COVID-19 cases on sequencing (genomic cluster 5); concordance between epidemiological and sequencing data |
| 36 | Indeterminate | Unvaccinated | 1 HCW on ward tested C+, 4 inpatient cases on ward; likely intra-ward transmission | Linked to other inpatient/HCW COVID-19 cases on sequencing (genomic cluster 5); concordance between epidemiological and sequencing data |
| 37 | Probable | Unvaccinated | 1 HCW on ward tested C+, 4 inpatient cases on ward; likely intra-ward transmission | Sequencing not performed due to low viral load |
| 38 | Indeterminate | Fully vaccinated | 3 inpatient cases on ward, likely intra-ward transmission | Linked to other inpatient COVID-19 cases on sequencing (genomic cluster 6); concordance between epidemiological and sequencing data |
| 39 | Probable | Fully vaccinated | 3 inpatient cases on ward, likely intra-ward transmission | Linked to other inpatient COVID-19 cases on sequencing (genomic cluster 6); concordance between epidemiological and sequencing data |
| 40 | Definite | Partially vaccinated | 3 inpatient cases on ward, likely intra-ward transmission | Sequencing not performed due to low viral load |
| 41 | Indeterminate | Unvaccinated | Shared cubicle with community-onset C+ case. | No sequencing link to other inpatient or HCW COVID-19 cases; **reclassified as** **plausible community-onset-case** |
| 42 | Indeterminate | Partially vaccinated | No epidemiological link to other inpatient or HCW COVID-19 cases | Sequencing not performed due to low viral load |
| 43 | Indeterminate | Fully vaccinated | Household cases C+ but also shared cubicle with community-onset C+ case. | No sequencing link to other inpatient or HCW COVID-19 cases; **reclassified as** **plausible community-onset-case** |
| 44 | Indeterminate | Fully vaccinated | No epidemiological link to other inpatient or HCW COVID-19 cases | No epidemiological or sequencing link to other inpatient or HCW COVID-19 cases; **reclassified as** **plausible community-onset-case** |
| 45 | Definite | Unvaccinated | No epidemiological link to other inpatient or HCW COVID-19 cases | No epidemiological or sequencing link to other inpatient or HCW COVID-19 cases. |
